# Atomistic basis of force generation, translocation, and coordination in a viral genome packaging motor

**DOI:** 10.1093/nar/gkab372

**Published:** 2021-05-29

**Authors:** Joshua Pajak, Erik Dill, Emilio Reyes-Aldrete, Mark A White, Brian A Kelch, Paul J Jardine, Gaurav Arya, Marc C Morais

**Affiliations:** Dept. of Mechanical Engineering and Materials Science, Duke University, Durham, NC 27708, USA; Dept. of Biochemistry and Molecular Biology, University of Texas Medical Branch, Galveston, TX 77555, USA; Dept. of Biochemistry and Molecular Biology, University of Texas Medical Branch, Galveston, TX 77555, USA; Sealy Center for Structural Biology and Molecular Biophysics, University of Texas Medical Branch, Galveston, TX 77555, USA; Dept. of Biochemistry and Molecular Pharmacology, University of Massachusetts Medical School, Worcester, MA 01605, USA; Dept. of Diagnostic and Biological Sciences, University of Minnesota, Minneapolis, MN 55455, USA; Dept. of Mechanical Engineering and Materials Science, Duke University, Durham, NC 27708, USA; Dept. of Biochemistry and Molecular Biology, University of Texas Medical Branch, Galveston, TX 77555, USA; Sealy Center for Structural Biology and Molecular Biophysics, University of Texas Medical Branch, Galveston, TX 77555, USA

## Abstract

Double-stranded DNA viruses package their genomes into pre-assembled capsids using virally-encoded ASCE ATPase ring motors. We present the first atomic-resolution crystal structure of a multimeric ring form of a viral dsDNA packaging motor, the ATPase of the asccφ28 phage, and characterize its atomic-level dynamics *via* long timescale molecular dynamics simulations. Based on these results, and previous single-molecule data and cryo-EM reconstruction of the homologous φ29 motor, we propose an overall packaging model that is driven by helical-to-planar transitions of the ring motor. These transitions are coordinated by inter-subunit interactions that regulate catalytic and force-generating events. Stepwise ATP binding to individual subunits increase their affinity for the helical DNA phosphate backbone, resulting in distortion away from the planar ring towards a helical configuration, inducing mechanical strain. Subsequent sequential hydrolysis events alleviate the accumulated mechanical strain, allowing a stepwise return of the motor to the planar conformation, translocating DNA in the process. This type of helical-to-planar mechanism could serve as a general framework for ring ATPases.

## INTRODUCTION

The Additional Strand, Conserved Glutamate (ASCE) superfamily is an ancient and ubiquitous class of NTPases, encompassing subfamilies such as AAA+ motors, RecA- and FtsK-like ATPases, and ABC transporters (1). These motors convert energy from NTP binding and/or hydrolysis into mechanical work, and typically perform biological segregation tasks such as proton transport, chromosomal segregation, DNA or RNA strand separation, and protein degradation. Double-stranded DNA (dsDNA) viruses, such as herpes-, adeno- and pox viruses, as well all tailed bacteriophages, encode for ASCE segregation motors that they use to package their genomes into preformed procapsids during virus replication (2–4). Determining the mechanistic aspects of viral DNA packaging motors will facilitate rational engineering of antibacterial phage therapeutics and microbiome manipulating agents, as well as provide attractive targets for anti-viral drug discovery (5,6). Further, among ASCE ATPases, viral packaging motors generate particularly high forces (>50 pN) to overcome the entropy loss, electrostatic repulsion and DNA stiffness that oppose DNA confinement (7–10). Thus, viral dsDNA packaging motors also provide a unique window into the mechanochemistry of force-generation found in this broad class of molecular motors.

The relatively small size and simplicity of φ29-like phages has facilitated advanced genetic, biochemical, and structural studies (3). For example, all components of the φ29 packaging system have been thoroughly characterized, and a robust highly efficient *in vitro* DNA packaging system has been developed (11). Furthermore, atomic resolution structures of all individual φ29 motor components are available (12–15) and medium resolution structures of motors assembled on capsids in various stages of assembly and/or packaging have been determined (15–19). These results indicate that the DNA packaging motor consists of a dodecameric portal protein, a pentameric prohead RNA (pRNA) and a pentameric ATPase (gene product 16; gp16) that assemble as co-axial rings at a unique vertex of the φ29 capsid.

Due in part to this experimental accessibility, single-molecule force spectroscopy (SMFS) experiments have provided valuable information on force-generation and dynamics of viral packaging motors. High-resolution measurements showed that the motor packages DNA in 10 bp ‘bursts’ comprised of four 2.5 bp sub-steps, each coupled to ATP hydrolysis and phosphate release. DNA translocation bursts are followed by a relatively long ‘dwell’ wherein DNA translocation pauses while each ADP is sequentially exchanged for ATP to reset the motor for the next burst (20–22).

Whereas genetic, biochemical, structural, and single-molecule studies have provided significant insights into the mechanochemistry of the φ29 packaging motor, the molecular basis of force-generation and coordination remains unresolved for any viral DNA packaging motor. Given the multi-component nature of the motor, it is difficult to determine how such coordination arises in the absence of high-resolution quaternary structural information. Likewise, it is difficult to determine the mechanisms of force generation in the absence of atomistic dynamic information. Hence, we determined the first high-resolution structure of a functional assembly of a φ29-like (asccφ28) ATPase and probed its dynamics *via* long-timescale molecular dynamics (MD) simulations. Together, these data resolve fundamental questions regarding inter-subunit coordination and force-generation, and enabled development of an atomistic model of viral DNA packaging wherein the motor transitions between helical and planar configurations to efficiently package DNA.

## MATERIALS AND METHODS

### Structure determination

#### Crystallization

Protein in buffer containing 50 mM sodium phosphate, pH 8.1, 400 mM sodium chloride, and 1 mM dithiothreitol was concentrated via filtration to 4.3 mg/ml. Several commercial sparse matrix screens were used to determine initial crystallization conditions, and initial crystals were obtained from the Wizard Classic screen (Rigaku) in 2 M ammonium sulfate, 0.1 M sodium citrate, pH 5.5 and Salt RX screen (Hampton Research) in 0.7 M citrate, 0.1 M Tris, pH 8.5. Gp11 has a predicted pI of 6.44, so would be positively or negatively charged, respectively, in the two conditions. Each condition served as the starting point for several optimization steps, and crystals exceeding 100μm were eventually grown in 24-well VDX trays by hanging-drop diffusion over 1000 μl of well solution. A rhombohedral crystal (Crystal Form A) was grown over wells containing 1.5–1.8 M ammonium sulfate, 0.1 M citrate, pH 5.3–5.7. The drop consisted of 1μl protein at 4.3 mg/ml mixed with 1 μl well solution containing 1.6 M ammonium sulfate, 0.1 M trisodium citrate, pH 5.7. The pH of trisodium citrate buffers was adjusted with hydrochloric acid. Crystals appeared after 2–4 days. Octahedral crystals (Crystal Form B) were grown from basic conditions containing 1.0 M trisodium citrate, 0.1 M Tris pH 8.3. The final pH of this well solution was measured at 8.9. Protein concentration was 3.1 mg/ml, and 1.5 μl of protein was combined with an equal volume of well solution. Both solutions were pre-chilled, and the tray was set up and incubated at 277 K. Well-formed crystals typically took more than a month to grow.

#### X-ray crystallographic data collection

Crystals of asccφ28 gp11 were soaked in mother liquor with 20% glycerol added as a cryoprotectant prior to flash-freezing. The X-ray diffraction data were collected at the Advanced Photon Source (APS) Life Science Collaborative Access Team (LS-CAT) beamlines (Supplementary Table S1). The data were indexed, scaled, and merged using HKL2000 (Supplementary Table S1) (23).

#### Structure solution and model building

The *P*3_2_21 structure, grown in 1.6 M ammonium sulphate and 0.1 M trisodium citrate, pH 5.7, was solved to 3.3 Å via SAD phasing using the programs Shelx (24) and HKL2MAP (25). The data were from a SeMet derivatized crystal. An initial model was built using a Parrot (26) NCS-averaged map in Buccaneer (27), both from CCP4 program suite (28). The final structure was refined to 2.9 Å using Phenix (29) and built in COOT (30). The P4_3_2_1_2 native structure, grown in 1.0 M trisodium citrate, 0.1 M tris (hydroxymethyl) aminomethane (Tris), pH 8.3, was solved using molecular replacement in Phaser (31), using the pentameric ring from the P3_2_21 cell as search model. The structure was refined using Phenix (29). Model-building was performed using COOT (30). The *P*4_3_2_1_2 Iodide-soaked structure was solved via direct phasing using the previous *P*4_3_2_1_2 structure described above as a starting model. The structure was refined using Phenix (29). Model-building was performed using COOT (30). Final refinement statistics for all structures are summarized in (Supplementary Table S1).

### Molecular dynamics simulations

#### Structure preparation

All molecular dynamics (MD) simulations of the pentameric ATPase ring of bacteriophage asccφ28 were started from the crystal structures reported herein. Missing side chains were added to the structures by using the Dunbrack rotamer library (32). The ‘ATP-bound’ pentamer was prepared by docking ATP into the binding pockets based on ATP-bound structures of homologous viral ATPases. Docking included ATP, Mg^2+^, and a water molecule situated between Mg^2+^ and the Walker B Asp, which is crucial for maintaining the canonical hydrogen bond coordination within the binding pocket. The ‘ADP-bound’ pentamer was prepared by removing the γ-phosphate from the initial coordinates of the ATP-bound subunits. The ‘4-ADP bound, 1-apo mixed occupancy’ pentamer was prepared by removing the unbinding ADP molecule from the final frame of the 5-ADP bound simulation. A 30 bp-long B-form double-stranded DNA molecule was generated by using the 3DNA webserver (33) and manually placed into the pore of the ATPase ring, which is large enough to accommodate B-form DNA with minimal steric clashes. The ‘ATPase-domain only’ pentamer was generated by truncating the C-terminal domains (residues 261 onwards) from the crystal structure, and a shorter 25 bp-long double-stranded DNA molecule was placed in the pore. Monomer simulations were started from one arbitrarily chosen subunit of the pentamer complex.

The pentamer of the P74–26 ATPase was constructed from the solved crystal structure of the ATPase domain P74–26 (PDB: 4ZNL) by using the M-ZDOCK protein-protein docking software (34), imposing five-fold symmetry. The structure produced matched the reported prediction (35), which was likewise generated with M-ZDOCK. ATP-bound structures were generated by placing Mg^2+^-ATP in the binding pocket according to the solved crystal structure of monomeric P74–26 ATPase containing bound ADP-BeF_3_ in the active site. As done for the asccφ28 pentamer, a double-stranded B-DNA molecule was generated with 3DNA and manually placed in the pore of the structure. Due to the larger size of the P74–26 ATPase, the molecule length was extended to 35 bp. After equilibration, the identified arginine finger was poised to catalyze hydrolysis in one and only one active site. To probe the effects of subsequent hydrolysis, ATP was removed from this active site, and the system equilibrated again. During this equilibration, the system transitioned from a planar ring to a helical arrangement, shearing at the single apo interface (Supplementary Figure S7).

Lastly, the D6E ATPase pentamer was constructed by superimposing a monomer structure (PDB: 5OE8) (36) which had undergone 2.4 μs of equilibrium MD simulation onto the asccφ28 crystal structure reported here. During the simulation, the monomer's lid subdomain extends away from the ATPase domain (Supplementary Movie S7), allowing for ample inter-subunit contacts similar to the asccφ28 crystal structure reported here. The C-terminal domain was truncated to prevent inter-subunit steric clash. A 25 bp-long double-stranded DNA molecule generated using 3DNA was manually placed into the pore of the structure.

#### Pentamer simulations

All-atom, explicit-solvent MD simulations of all pentamers were carried out using the Anton 2 simulation package and supercomputer (37). The equilibration runs, including structure preparation and energy minimization, were all performed using the Amber18 simulation package with GPU optimization (38), as all systems must be equilibrated before production runs can be performed on Anton 2. The equilibration and production simulations both used the AMBER ff99SB-ILDN force field to describe protein interactions (39), and the bsc1 AMBER parameter update to describe DNA interactions (40). ATP and ADP parameters are taken from the AMBER parameter database (41).

For equilibration using Amber18 package, all systems were centered in a periodic cubic box of TIP3P water with 14 Å minimum padding. The systems were charge-neutralized with counterions and additional salt was added to reach 150 mM concentration. The systems were energy minimized using a combination of steepest descent and conjugate gradient, and then slowly heated in the NVT ensemble from 100 to 310 K over 100 ps using the Langevin thermostat. Bonds connecting heavy atoms to hydrogen were constrained through the SHAKE algorithm, and a 2-fs integration time step was used to propagate the equations of motion. Subsequently, each system was subjected to short simulation in the NPT ensemble, held at 310 K via the Langevin thermostat and 1 bar with the Monte Carlo barostat to equilibrate the density. Finally, each system was simulated for at least 2.4 μs on the Anton 2 supercomputer. These production runs were performed in the NPT ensemble held at 310 K and 1 bar using the Nosé-Hoover thermostat and the MTK barostat and a 2.5 fs integration time step to propagate the equations of motion.

#### Characterizing subunit motions and flexibility

Principal components (PCs) and root-mean-square fluctuations (RMSF) were calculated for each subunit individually by aligning the trajectories to minimize root-mean-square deviation (RMSD) of that single subunit. Thus, the PCs and RMSF represent internal degrees of freedom of a subunit and do not account for motion of a subunit relative to the rest of the pentamer. These quantities were calculated on a per-residue basis using the α-carbons in each residue excluding the side chain. Thus, the obtained values reflect backbone motion and not rotamer states. PCs and RMSF were calculated using ProDy software as interfaced with VMD. RMSF curves in Figure 5 were calculated by averaging the per-residue RMSF of all five subunits within a single trajectory, and the uncertainty reported is the standard error of the mean.

Subunit variation (Figure 5 and Supplementary Figure S6) was determined by calculating the residue-residue pairwise distances within a single subunit for each subunit of a given pentamer structure. Then the five values were averaged, and the standard deviation of this average is plotted as heat maps in Figure 5. High standard deviation in the average residue-residue pairwise distance indicates structural variability across the five subunits.

#### Monomer simulations

To better understand the dynamics of an individual subunit within the pentamer, we also performed 100 ns long all-atom explicit MD simulations of a monomer in the apo, ATP-bound, and ADP-bound states. These short timescale monomer simulations were performed exclusively in the Amber18 simulation package with GPU acceleration. We performed simulations using both the AMBER ff99SB-ILDN/TIP3P and the AMBER ff19SB/OPC protein/water force field combinations. The reason for considering two separate force field combinations is provided below. These systems were centered in a periodic truncated-octahedron box of water to reduce the total number of particles and save computation time. The systems were equilibrated using the same procedure described above for pentamers. 100-ns production runs were performed in triplicate for both set of force fields in the NPT statistical ensemble, held at 310 K and 1 bar by the Langevin thermostat and Monte Carlo barostat.

The AMBER ff99SB-ILDN/TIP3P simulations allowed for direct comparison to the pentamer simulations performed on Anton 2 which used the same pairing of force fields. However, it has been shown that the TIP3P water model under-predicts the strength of protein-water interactions and promotes compact secondary structure formation. We were primarily interested in the dynamics of the lid subdomain, which is extended away from the ATPase domain via a flexible linker. In the pentamer structures, the lid subdomain forms extensive contacts with neighboring subunits, which may help stabilize it in its extended state. In the monomer simulations these protein-protein contacts are replaced by protein-water contacts. Thus, we also considered the new AMBER ff19SB/OPC force field pairing (42,43), which strengthens the protein-water interaction and helps stabilize extended states. In the end, both force field pairings predicted similar conformations and dynamics of the lid subdomain on the timescale we considered.

**Figure 1.**
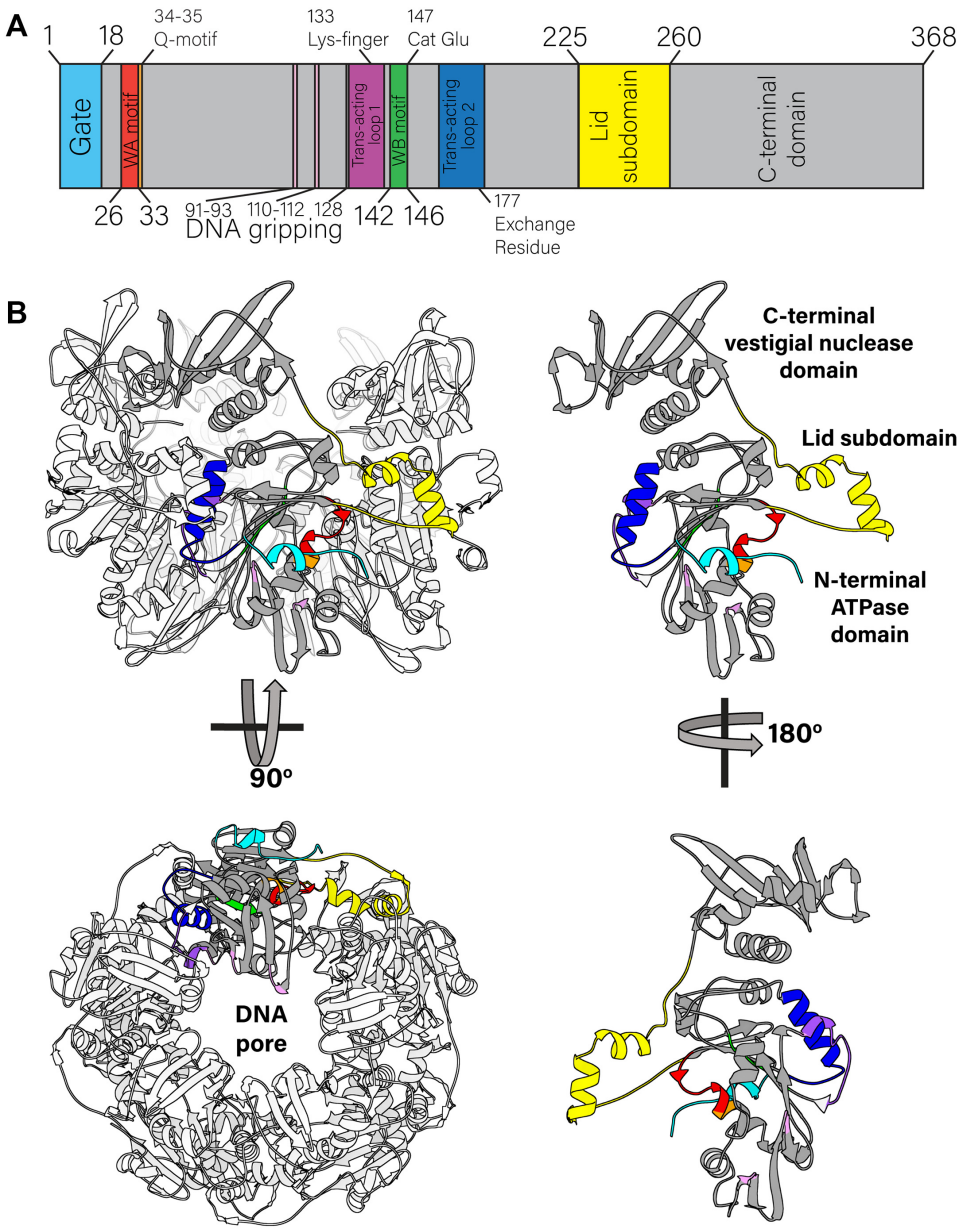
Quaternary and tertiary structure of the asccφ28 packaging ATPase. (**A**) Sequence of the packaging ATPase contains canonical ASCE motifs, such as the Walker A and Walker B motifs. (**B**) Structure of the pentamer (left), with a single subunit highlighted in gray and its lid subdomain highlighted in yellow is shown from side and end-on views (top and bottom panels, respectively). The lid subdomain mediates most of the inter-subunit contacts. The monomer in isolation (right) is color coded to match the motif classification in panel A, and is shown in two side views, from the exterior of the motor (top) and the interior of the lumen (bottom).

**Figure 2.**
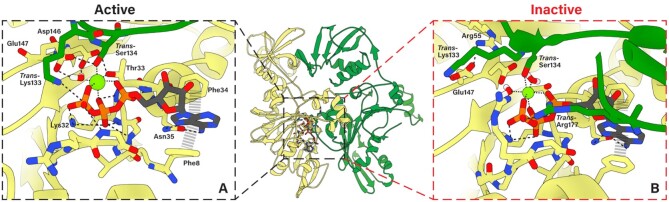
ATP-binding poses predicted from MD simulations. (**A**) Active ATP-binding pocket contains canonically predicted interactions. The cis-acting (yellow) subunit's Walker A motif backbone NH groups bind the β-phosphate, Lys32 binds the γ- and β-phosphates, and Thr33 chelates Mg^2+^. Downstream of the Walker A motif, Phe34 and Asn35 function as the Q-motif, and bind the adenosine with help from N-terminal gate Phe8. The Walker B motif Asp146 and catalytic Glu147 isolate a single water molecule, which chelates Mg^2+^ (green sphere). Residues donated in trans from the neighboring subunit (green) also participate in ATP-binding. Notably, Lys133 is seen interacting with the γ-phosphate and Ser134 chelates Mg^2+^. (**B**) Inactive pose maintains many of the same interactions as the active pose, with a few key differences. Arg177 is now donated in trans to interact with the γ-phosphate, while Lys133 interacts with catalytic Glu147 away from the γ-phosphate. This interaction is stabilized by cis-acting Arg55.

**Figure 3.**
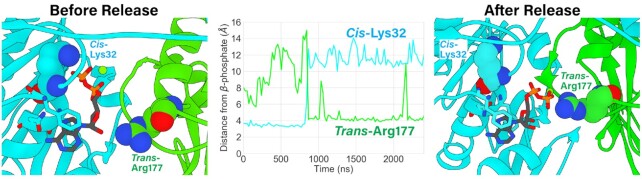
ADP-release is promoted by a trans-acting exchange residue. ADP unbinding is characterized by dissociation of the β-phosphate from the *cis*-acting Walker A Lys32 (interaction shown left panel) and concomitant association (time-evolved distance shown middle panel) of the β-phosphate to *trans*-acting Arg177 (interaction shown right panel), which is implicated as being the exchange residue. Residues interacting with the adenosine (Phe8, Phe34, Asn35, shown unlabeled) largely maintain their interactions, acting as a pivot to remove the phosphates from the binding pocket. The *cis*-acting Lys32 and *trans*-acting Arg177 are shown as spheres and are labeled. The *cis*-acting enzyme is light blue, and the *trans*-acting enzyme is green.

**Figure 4.**
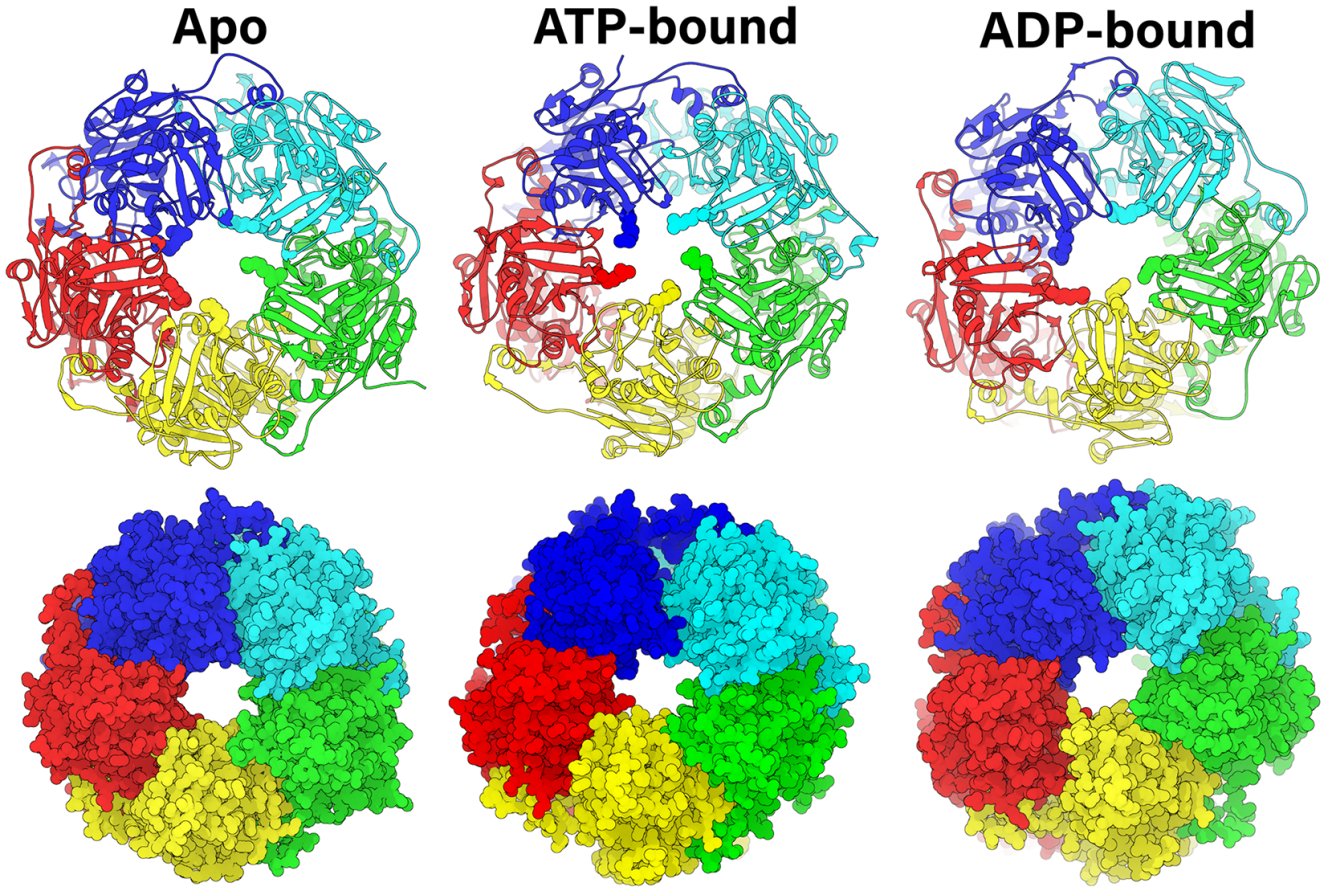
Pore geometry is modulated by nucleotide occupancy. Pentamer complex simulated in apo, ATP-bound, and ADP-bound states with substrate DNA (not shown for easier visualization) as Richardson diagrams (top row) and space-filling representations (bottom row). In the Richardson diagrams, the five Arg110s are shown as spheres to highlight their contribution towards DNA gripping in the ATP-bound state. The pore is less constricted in the apo and ADP-bound states than the ATP-bound state.

**Figure 5.**
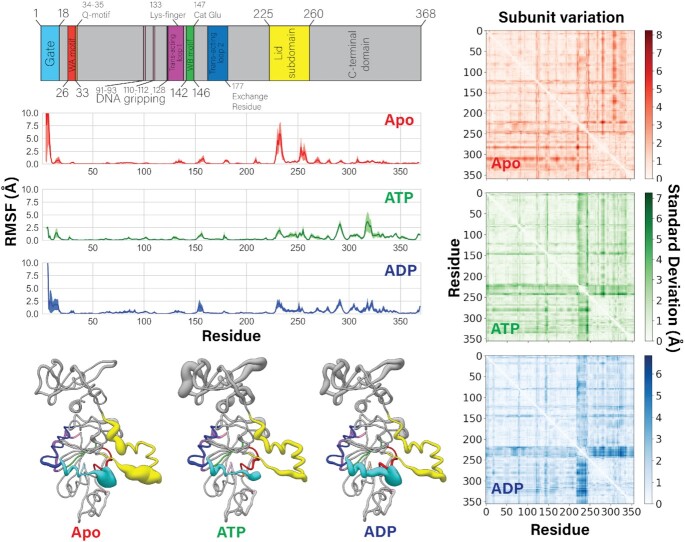
Flexibility of subunits is modulated by nucleotide occupancy. (Left column) Motif identity chart is reproduced at the top for easy reference. Averaged root-mean-square fluctuations (RMSF) of the ATPase alpha carbons over 2.4 μs of MD simulations are plotted. The standard error of the mean is shaded in each plot. Beneath the plots, the average RMSF value is coded to the radius of a worm-representation of the enzyme, which is color coded to the sequence. Thicker radius indicates higher flexibility. The apo state has a significantly more flexible lid subdomain than the ATP- or ADP-bound monomers. (Right column) Intra-subunit inter-residue distances are calculated in the apo, ATP-, and ADP-bound simulated states. The results from each of the five subunits are averaged. The standard deviations of the average are plotted as heatmaps, indicating structural variation across subunits within the pentamer. The ATP- and ADP-bound monomers have a band of high standard deviation in the lid subdomain (residues ∼225–245), indicating that although each lid subdomain is rigid, they are in different poses. This band is muted in the apo state, indicating that the average positions of the lid subdomains are roughly equivalent, despite their flexibility. These observations correlate well with the asymmetry of the ATP- and ADP-bound pentamers, and the symmetry of the apo pentamer, given that the lid subdomain mediates most of the inter-subunit contacts.

**Figure 6.**
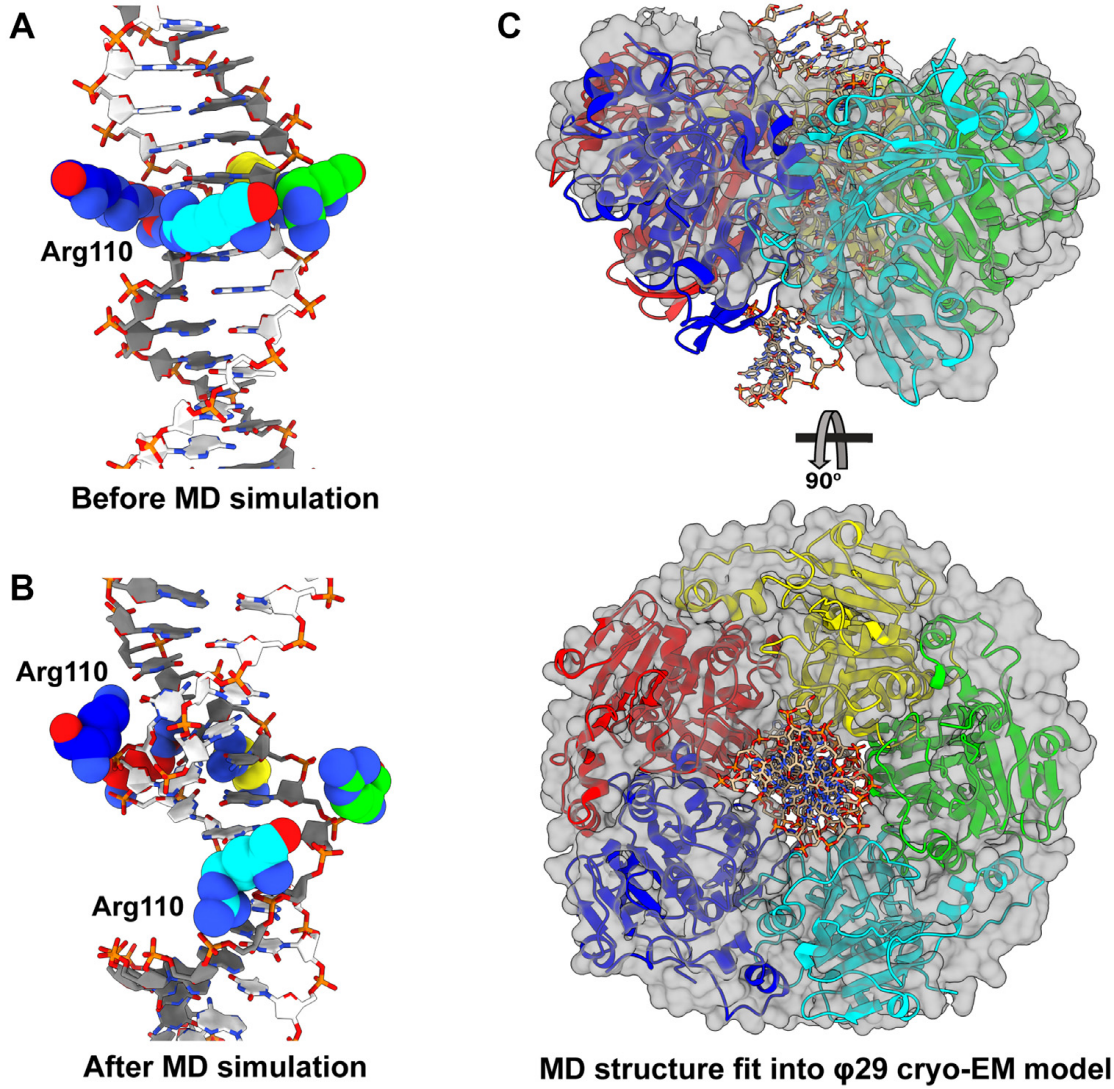
Predicted helicity of asccφ28 ATPase domains. (**A**) Initial set up of the asccφ28 NTD ring with DNA in the pore, prior to MD simulation. Initially, all five Arg110s are in a planar ring. (**B**) The predicted configuration after MD simulation. The five Arg110s adopt a helical pitch complementary to DNA, primarily tracking one strand of DNA. The Arg110s approach the phosphates differently: the lower three subunits (cyan, green, yellow) track ‘above’ the dark gray strand every two base pairs. The remaining two subunits (red, blue) fit into the minor groove and contact the dark gray strand from ‘below’, while also interacting with nucleobases. (**C**) The MD simulated asccφ28 NTD ring was fit into the cryo-EM reconstruction of φ29 stalled during packaging. The asccφ28 ring is shown as Richardson diagrams, DNA as sticks, and the model of φ29 NTDs built into the reconstruction as a translucent surface.

SAMSON (44) was used to create Figures 1–4. UCSF Chimera (45) was used to visualize Figure 5. UCSF ChimeraX (46) was used to visualize Figure 6.

## RESULTS

Despite extensive efforts, it has not been possible to assemble the functional ring-form of the packaging ATPase from bacteriophage φ29 detached from procapsids. This is not surprising, since the φ29 ATPase only assembles functional rings by virtue of binding to the procapsid (18). In contrast, a close homolog of the φ29 ATPase from the related bacteriophage asccφ28 (gp11) has been shown to form highly soluble functional rings; extensive analytical ultracentrifugation and small-angle X-ray scattering experiments show that the ATPase forms pentameric rings in solution (47). The 45% sequence similarity between the two proteins assures their structures will be nearly identical. Thus, we used asccφ28 gp11 for crystallographic structure determination.

### X-ray crystal structure determination

Cloning, protein purification, kinetic analysis, AUC, SAXS, negative stain TEM and preliminary crystallization of gp11 has been previously reported (47). Two crystal forms of gp11 were obtained: (i) tetragonal crystals belonging to space group *P*4_3_2_1_2 and (ii) trigonal crystals belonging to space group *P*3_2_2_1_. Single-wavelength anomalous dispersion (SAD) was used to obtain experimental phases for the *P*3_2_2_1_ crystals grown from selenomethionine labeled protein (PDB 7JQ6; Supplementary Table S1). The final refined structure was used as a molecular replacement search model to phase the data from the *P*4_3_2_1_2 space group (PDB 7JQ7, 7JQP).

### Quaternary structure of gp11

Both the tetragonal and trigonal crystal forms had similar pentameric rings in their crystallographic asymmetric units despite having substantially different packing environments (Figure 1), indicating that the observed pentamer is the biological assembly. This stoichiometry is consistent with previously reported biochemical and biophysical analysis that indicated gp11 forms pentameric rings in solution (47). Of note, the kinetic parameters of ATP binding and hydrolysis by isolated gp11 rings are similar to the parameters obtained for φ29 and other bacteriophage DNA packaging motors, but only once these other ATPases are assembled as functional rings on their respective procapsids (47). In the absence of procapsids, other packaging motors negligibly hydrolyze ATP, presumably since they are in monomeric form and therefore cannot efficiently bind or hydrolyze ATP (see also below). Additionally, the arrangement of the subunits is similar to the recent structure of the φ29 particles stalled during packaging (19), with the notable difference that the φ29 ATPase structure is helical rather than planar. Hence, the pentameric stoichiometry of gp11 observed here likely reflects a functional assembly during DNA packaging.

### Tertiary structure of gp11

Individual subunits within the ring are organized into two globular domains connected by a linker domain, each of which is nearly identical to the corresponding domains of the φ29 gp16 (Supplementary Figure S1) (19). Like φ29 gp16, the N-terminal domain adopts the canonical ASCE ATPase fold, while the C-terminal domain is a ‘vestigial nuclease domain’ (13). The linker domain (residues 225 to 260) adopts a helix-loop-helix fold, again like φ29 gp16 and reminiscent of the lid subdomain identified in other ASCE ATPases such as AAA+ and helicases (1).

### ATPase active site: *cis*-contributions

While the three structures described here were determined in the absence of nucleotide, the active sites of ASCE ATPases are well characterized and can be accurately identified. In the solved structures, the ASCE ATPase active site is situated between two subunits, such that both subunits participate in catalysis. The *cis-*acting side of the ATP-binding interface resides on one edge of the central beta-sheet of the Rossmann fold and includes canonical Walker A (26-GGRGVGKT-33) and Walker B (142-YLVFD-146) motifs.

Two distinct conformations of the Walker A motif are apparent in our crystal structures (Supplementary Figure S2). One conformation binds either a SO_4_^2−^ or PO_4_^3−^ ion in the canonical β-phosphate position. The ion forms hydrogen bonds with the backbone of the Walker A motif in lieu of a β-phosphate, typically provided by ATP or ADP, which explains why the Walker A backbone adopts a conformation similar to a typical nucleotide-bound ATPase configuration. However, because the structure was solved in absence of nucleotide or nucleotide analog, it lacks any interactions mediated by the α- and γ-phosphates, ribose sugar, or adenosine base. Thus, this structure likely represents a conformation with mixed characteristics from both the apo and nucleotide-bound configurations.

In contrast, the conformation observed in the iodine-bound structure shows that an iodine atom sits in a hydrophobic pocket that is occupied by Val30 in the other structures. As a result of displacing Val30, the Walker A motif adopts an extra helical turn in the phosphate-binding loop. This conformational change excludes the possibility of binding nucleotide since: (i) the sidechain of Val30 is in the center of the active site, occluding nucleotide, and (ii) the Walker A backbone amide groups are repositioned such that they can no longer hydrogen bond with the β-phosphate. Initially, we assumed that the nucleotide-blocking valine was an artifact of heavy atom derivatization. However, subsequent structural analysis and MD simulations indicated that the two conformations of this loop may reflect an ability to switch between nucleotide accepting and occluding conformations, providing a mechanism for regulation of ATP binding and ADP release (see below).

### ATPase active site: *trans*-contributions

The *trans*-acting side of the subunit interface consists primarily of two helical segments (residues 129–139 and 161–178) that reside on the side of the central Rossmann fold opposite the *cis*-contributing elements. These helices position polar- and positively-charged residues in the ‘neighboring’ active site that contribute to ATP binding and hydrolysis, as well as to phosphate and ADP release (Figure 1). Notably, Arg177 is positioned in the active site of all three crystal structures. Arg177 in asccφ28 corresponds to Arg146 in the φ29 ATPase, which had previously been identified as a *trans*-acting arginine finger (14,48). Arginine fingers are ubiquitous in ring ATPases and are generally believed to catalyze ATP hydrolysis by stabilizing the transition state (49). However, the sidechain of Arg177 is not optimally positioned to coordinate the expected position of the γ-phosphate of ATP (Supplementary Figure S2). Thus, for Arg177 to act as the arginine finger and catalyze hydrolysis *in trans*, the interface would require significant rearrangements.

A different positively charged residue, Lys133, is better positioned to interact *in trans* with the expected position of the γ-phosphate of ATP (Supplementary Figure S2). Such interaction would require significantly fewer structural rearrangements. Further, Lys133 corresponds to the arginine finger identified in the bacteriophage P74–26 packaging ATPase , indicating that Lys133 may function as a ‘lysine finger.’ We note that the use of a lysine to catalyze hydrolysis *in trans* instead of the ‘traditional’ arginine finger has been observed in both RecA (50) and DnaC (51) ATPases, and thus there is precedence for a lysine finger in ATPase ring motors.

### Phosphate binding and regulation of hydrolysis

To further understand how subunits around the ring bind ATP, and how this binding might modulate DNA gripping, we performed long timescale MD simulations of the ATPase ring. For the starting structure, Mg^2+^-ATP was positioned into each subunit according to the structure of the BeF_3_-ADP-bound phage P74–26 packaging ATPase . Additionally, a 30-bp, B-form dsDNA was placed in the central pore of the pentamer. The structure was equilibrated and its equilibrium dynamics were sampled *via* MD simulations for 2.4 μs. The simulations predicted that Mg^2+^-ATP binds to the *cis*-acting side of the inter-subunit active site *via* canonical interactions with the Walker A motif: (i) the β-phosphate of ATP forms several hydrogen bonds with the backbone nitrogens of the Walker A motif; (ii) the critical P-loop Lys32 coordinates the β- and γ-phosphates and (iii) Thr33 chelates the Mg^2+^ ion (Figure 2A). The *cis-*acting Walker B motif residues engage in similar conserved canonical interactions: (a) Asp146 chelates the Mg^2+^ ion through a water molecule and (b) Asp146 hydrogen bonds to the Walker A Thr33, which has been previously predicted to be a key interaction that helps close the active site as part of the tight-binding transition (52,53).

While the *cis*-acting interactions were expected, the simulations further predicted that the *trans*-acting residues donated from the neighboring subunit form a tight hydrogen bonding network with the *cis*-acting Walker motifs that is centered around the γ-phosphate (Figure 2A). *Trans*-acting Lys133 interacts with the γ-phosphate of ATP, consistent with Lys133 functioning analogously to ‘arginine fingers’ described in other systems. Such an interaction would help polarize the P–O bond, and stabilize the negative charge on the transition state (49). Further, the residue immediately downstream of Lys133, Ser134, chelates the Mg^2+^ ion *in trans*. This lysine-serine pair is distinct from SRC motifs found in many AAA+ motors that contain an arginine finger (54). In the AAA+ SRC motif, the serine residue is upstream of the *trans*-acting catalytic residue, and does not chelate Mg^2+^. Thus, to the best of our knowledge, our structure and simulations predict a new motif for *trans*-activated catalysis in ASCE enzymes.

The simulations also predicted that the *cis-*acting Walker B catalytic Glu147 hydrogen bonds to this *trans*-acting Ser134. The consequence of these interactions is a tight hydrogen bonding network centered around a single water molecule. The hydrogen atoms of this caged water hydrogen bond with the oxygens on the Walker B 146-DE-147 carboxylate groups, whereas the oxygen atom chelates the Mg^2+^ ion. Such a configuration would polarize electron density onto the oxygen atom. Hence, the water molecule is primed for deprotonation by Glu147, with the resulting nucleophilic hydroxide ion poised for attack at the γ-phosphate as described above. It is interesting to consider the conservation of the DE pair in light of our structures and simulation; the geometry of the active site ensures that the glutamate is positioned such that deprotonation leaves the lone pair of electrons on the nucleophilic hydroxide pointing directly at the phosphate target. If the positions of Asp and Glu were switched, the hydroxide ion would not be optimally oriented for nucleophilic attack. Indeed, DE switch mutations in viral packaging ATPases typically abrogate ATPase activity (55).

In addition to the hydrolysis-competent active site described above, our simulations suggests that the binding interface can also adopt an ‘inactive’ conformation. This conformation positions the catalytic Glu147 carboxylate group away from the γ-phosphate and towards *cis*-acting Arg55. We also found that while Ser134 still chelates Mg^2+^*in trans* in the inactive conformation, Lys133 no longer interacts with the γ-phosphate and instead interacts with Glu147, helping Arg55 stabilize the inactive pose of the binding interface. The resulting interaction between Glu147 and Arg55 is analogous to the ‘glutamate switch’ interaction found in AAA+ enzymes, and is indicative of a binding interface that is catalytically incompetent (56). In these other systems, the glutamate switch regulates the timing of hydrolysis, thus we suspect a similar role in viral DNA packaging motors. Indeed, a similar interaction is found in crystal structures of the φ29 and Sf6 packaging ATPases with bound nucleotide (Supplementary Figure S3).

### Adenosine binding and control over active site accessibility

The adenosine base of ATP binds similarly in both the ‘inactive’ and ‘active’ interfaces (Figure 2A, B). An aromatic residue immediately downstream of the canonical Walker A motif, Phe34, π-stacks with the adenosine base. The adenosine forms bidentate hydrogen bonds with the next residue, Asn35. A pairing of an aromatic residue followed by a carboxamide has been previously identified as the ‘Q motif’ in DEAD-box RNA helicases and viral packaging ATPases. The pair's function was implicated in binding the adenosine ring in the λ phage packaging ATPase (57).

On the other side of the adenosine base, the N-terminal loop repositions itself so that Phe8 π-stacks on the side of the adenosine opposite of Phe34. This contrasts with the position of the N-terminal loop in the crystal structure, which positions Phe8 farther away from the Walker A motif. Thus, the N-terminal loop forms a gate that can either open to allow nucleotide exchange, or close to tightly bind ATP. Similar π-stacking sandwiches flanking either side of adenosine have been observed in solved structures of other ATPases, such as *E. coli* MutS and the Human Catalytic Step I Spliceosome (58,59). This suggests that similar active-site gating mechanisms may be present in other ring ATPases. In the closely related bacteriophage φ29, the N-terminal loop makes critical contact with pRNA (19). Although gp11 was solved in absence of pRNA, the positions of all pRNA-contacting residues are conserved. Thus, in φ29-like phages, pRNA may play a role in nucleotide binding/release by affecting the position or dynamics of the gate motif.

### ADP release is promoted by a *trans*-acting arginine

The ATP-bound MD simulations described above show how individual subunit interfaces can bind and hydrolyze ATP. As revealed by SMFS studies, after all five ATPs have been hydrolyzed during the DNA translocating burst, the motor coordinates exchange of ADP for ATP in a sequential and interlaced manner (20). To begin to understand the atomistic basis of these events, we simulated the structure of gp11 with ADP bound in each active site. The system was equilibrated with bound ADP and its equilibrium dynamics were simulated for at least 2.4 μs. Over this long sampling period, we were able to capture the initial steps of ADP-release. The α- and β-phosphates of ADP detached from the Walker A phosphate-binding motifs, while adenosine interactions were maintained. Importantly, release of the β-phosphate by the Walker A Lys32 was observed to be concomitant with interaction of the β-phosphate with the *trans*-acting Arg177 (Figure 3, Supplementary Movie S1). Thus, our simulations indicate that this arginine functions to promote nucleotide exchange, rather than as the canonical catalytic arginine finger. This observation provides atomistic explanation for recent SMFS observations that suggested a role for the analogous φ29 gp16 Arg146 in nucleotide exchange (48).

After the phosphates were released from the Walker A motif, the Walker A backbone amide groups repositioned such that they could no longer bind the β-phosphate of ATP/ADP (Supplementary Movie S2). This prevented ADP from accessing energetically favorable hydrogen bonding with the Walker A motif, reducing overall nucleotide affinity and likely precipitating complete dissociation of ADP from the active site. While our simulations were longer than typical MD simulations, they were not long enough to predict diffusion of ADP into solution. Nonetheless, we were interested in understanding further structural rearrangements that might occur upon complete dissociation of ADP. Thus, we performed additional simulations to probe the conformation and dynamics of a 4-ADP-bound, 1-apo subunit motor by removing the partially-unbound ADP described above from the system. We observe that the apo subunit's Walker A motif backbone continued to rearrange, and finally positioned the Walker A Val30 in the ‘blocking’ pose observed in the iodine crystal structure described above (Supplementary Movie S3). This phenomenon has been characterized in myosin, where propensity of the Walker A motif to adopt a pose that is not receptive to β-phosphate hydrogen bonding is reported to be a predictor of ADP release rates (60). Thus the simulations suggest that this pose may not be an artifact caused by iodine binding, but rather may be a mechanism that regulates ADP release and subsequent ATP binding.

### Positively-charged residues line the DNA pore

The crystallographic structures show that the pore through which DNA translocates is lined with several positively charged residues (Supplementary Figure S4). In the C-terminal domain, Lys332 and Lys336 point directly toward the channel lumen, and are thus well positioned to interact with DNA. A slight rotation of the C-terminal domain relative to the N-terminal domain would cause these side-chains to rotate out of the channel center and position Lys333, Lys334 and Lys366 in the lumen. Hence, both sets of residues may play a role in the packaging process, though in different stages of the mechanochemical cycle. This possibility is supported by the cryo-EM structure of φ29 particles imaged during packaging, which showed that residues approximately equivalent to Lys333, Lys334 and Lys366 interact with the substrate DNA during the dwell phase, presumably to prevent DNA slippage while the motor resets and exchanges ADP for ATP (13,19). Hence, these residues likely function similarly in asccφ28.

The N-terminal (ATPase) domain also donates positively charged residues into the pore of the pentameric ring, namely Lys66, Lys92, Lys107, Arg110 and Arg128 (Figure 1). While N-terminal domain-DNA interactions will be described in greater depth below, it is worth noting that these residues are well-conserved among other viral packaging ATPases, suggesting a conserved function (Supplementary Table S2). Of note, asccφ28 Arg110 in is the only strictly conserved positively charged residue in all solved dsDNA viral packaging ATPases (Supplementary Table S2). It was previously shown that the analogous Arg101 from the bacteriophage P74–26 packaging ATPase is absolutely necessary to bind substrate DNA (61), suggesting a direct role in DNA translocation. The position of this residue in the interior of the pore supports this assignment.

### DNA-ATPase interactions depend on nucleotide-bound state

It is well known that the affinity for biopolymer substrates in ring ATPases depends on whether the ATPase active site is empty or if it has ATP or ADP bound. However, the structural basis for these changes in affinity is poorly understood. Thus, to assess the motor's nucleotide-dependent affinity for substrate DNA, we systematically compared the DNA-binding residues from the ATP- and ADP-bound simulations described above (which included DNA in the pore). To complete the comparison, we performed equivalent 2.4 μs simulations of an apo pentamer with substrate DNA positioned in the pore. We observed that both the orientation of the positively-charged residues and the overall shape of the pore depend on nucleotide occupancy of the ATPase interfacial active sites.

Amongst the positively charged residues located in the lumen, we find that Arg110 is the most directly affected by nucleotide occupancy (Figure 4). In the all-apo simulation, all but one Arg110 lay flat along the interior of the pore, and do not interact strongly with DNA. Similarly, in the ADP-bound simulation, all of the Arg110s lay flat against the pore. On the other hand, when all five interfaces are bound with ATP, Arg110 is re-positioned into the pore as ‘prongs’ that help grip DNA tightly. Thus, it appears that the presence of the γ-phosphate of ATP actuates the DNA-gripping signal, as has been predicted in viral packaging ATPases (62). This observation helps explain why an arginine at this position is highly conserved and provides new insight into the general phenomenon of ATP-dependent increase in motor affinity for biopolymer substrates.

### Lid subdomain flexibility provides molecular basis of force generation and motor reset

To quantitatively characterize the dynamics of the asccφ28 packaging ATPase obtained from our equilibrium MD simulations, we performed principal component analysis (PCA) coupled with root-mean-square fluctuation (RMSF) calculations on the alpha carbons of every residue in the gp11 ring in the different nucleotide bound states. RMSF provides us with a measure of how flexible a residue or motif is, while PCA allows us to understand whether flexibility is correlated as concerted motion along a specific direction or if the motion is essentially random (Figure 5 and Supplementary Figure S5).

Our analysis shows that the apo state is characterized by highly flexible N-terminal gate and lid subdomains. Flexibility of the lid subdomain can be attributed to the coils connecting the lid subdomain to the the N- and C-terminal domains. Thus, the helix-loop-helix motif of the lid subdomain moves as a rigid body, allowing it to maintain inter- subunit contacts with, and impart force on, its neighboring subunit.

Upon ATP binding, the simulations predict that both the N-terminal gate and the lid subdomains lose flexibility, and that the first principal component of motion is a rotation of the lid subdomain towards the ATPase active site (Supplementary Figure S5). Because the lid subdomain largely mediates inter-subunit contacts, lid subdomain rotation would pull two subunits closer together, enabling *trans*-acting catalysis; the mechanistic implications of this rotation are described below. In contrast, the ADP-bound state on average is characterized by a highly flexible N-terminal gate motif, but a rigid lid subdomain. However, the subunit wherein we observe ADP release has a concerted rotation of the lid subdomain away from the ATPase active site (Supplementary Figure S5). This again suggests that ATP binding/ADP unbinding causes rotation of the lid subdomain. The dynamics described above in the pentamer simulations are echoed by similar dynamics observed in short-timescale simulations of a single subunit in the apo, ATP-bound, and ADP-bound states (Supplementary Movies S4, S5, S6), which show that nucleotide binding rotates the lid subdomain over the ATPase active site.

To identify other regions of the protein that might be affected by lid subdomain rotation, we calculated intra-subunit residue-residue pairwise distances for all five subunits, and plotted the standard deviation of the average distance (Figure 5). High standard deviation of average residue-residue distances indicates that the relative positions of residues vary across the five subunits; low standard deviation indicates structural homogeneity. The region of highest-standard deviation observed in the ATP- and ADP-bound simulated states corresponds to the lid subdomain. Likewise analysis of the three crystal structures shows that most of the variance is concentrated in the lid subdomain (Supplementary Figure S6). There is a second band of significant variation, which corresponds with the *trans*-acting lysine finger and its adjacent residues (residues 130–133) (Figure 5, Supplementary Figure S7). Thus, not only does the lid subdomain directly contact *trans-*acting residues (Figure 1), this analysis further indicates that the lid subdomain can modulate the positions of the key catalytic *trans*-acting residues. In summary, nucleotide-actuated rotation of a subunit's lid subdomain can rearrange the motor's overall quaternary structure and finely tune the position of *trans*-acting catalytic residues for appropriate function.

### Helix tracking in viral DNA packaging ATPases

We recently solved the structure of the φ29 packaging ATPase attached to the prohead and stalled during packaging (19). This asymmetric cryo-EM reconstruction showed that the N-terminal domains of the ATPase ring adopt a helical conformation as each subunit tracks the substrate DNA. However, the crystal structure of asccφ28 gp11 ring reported here shows no such helicity in the N- or C-terminal domains. Likewise, the above MD simulations do not predict that the ATPase ring adopts a helical conformation. We suspect that we do not observe helicity in the crystal structures due to lack of substrate DNA imparting helicity. Lack of helicity in the MD simulations is likely attributable to interactions between the N- and C-terminal domains that result in a large activation energy barrier between extended and compact conformations. Thus, this transition may be kinetically infeasible to sample on the microsecond timescale of our simulations.

To remove this barrier, we simulated a pentamer ring composed of truncated monomers which contain only the N-terminal and lid (sub)domains (residues 1–260) (Figure 6A). Similar truncations in AAA+ systems have helped reveal biologically relevant helical conformations in cryo-EM structures (63,64). MD simulations of the truncated ATPase assembly suggested that the motor can transition between planar and helical arrangements, such that each subunit interacts with the helical phosphate backbone of one strand of DNA (Figure 6B). The helical arrangement of the Arg110s agrees with both the placement of an analogous DNA-gripping residue observed in the cryo-EM structure of φ29^20^, and SMFS experiments that showed the motor primarily tracks along a single strand of DNA (65). Morphs between the two conformations can be seen in Supplementary Movies S8 and S9. To test if this propensity of ATPase domains to track DNA could be a general feature of viral packaging ATPases, we simulated pentamer assemblies of the P74–26 and D6E packaging ATPases constructed *in silico*. Again, our simulations predicted that these structures transition from the starting planar ring configuration to a helical configuration (Supplementary Figure S7). Furthermore, the simulated structures of the three systems (asccφ28, P74–26, and D6E) fit well into the cryo-EM reconstruction of the φ29 motor stalled with ATP analog (19) (Figure 6C, Supplementary Movies S10–S12).

## DISCUSSION

An emerging feature of ring ATPases is that subunits in the ‘ring’ arrange themselves as a helix as they track their biopolymeric substrates (64,66–69). Of particular relevance, a cryo-EM structure of φ29 stalled during packaging showed that its packaging motor adopts a helical pitch complementary to the double-stranded DNA substrate (19). Based on this observation and the well-characterized behavior in SMFS studies (20–21,70–71), it was proposed that φ29-like packaging motors transition between helical and planar states to translocate DNA. While the SMFS data identified the intermediate states that define the mechanochemical cycle and the cryo-EM structure documented the helical end state of the cycle, these data only tell half the story. The structures and simulations described here tell the other half of the story by documenting the structure of the planar end state and revealing the molecular basis of the conformational changes that drive the mechanochemical cycle.

### Helical-to-planar ratchet mechanism

**Figure 7.**
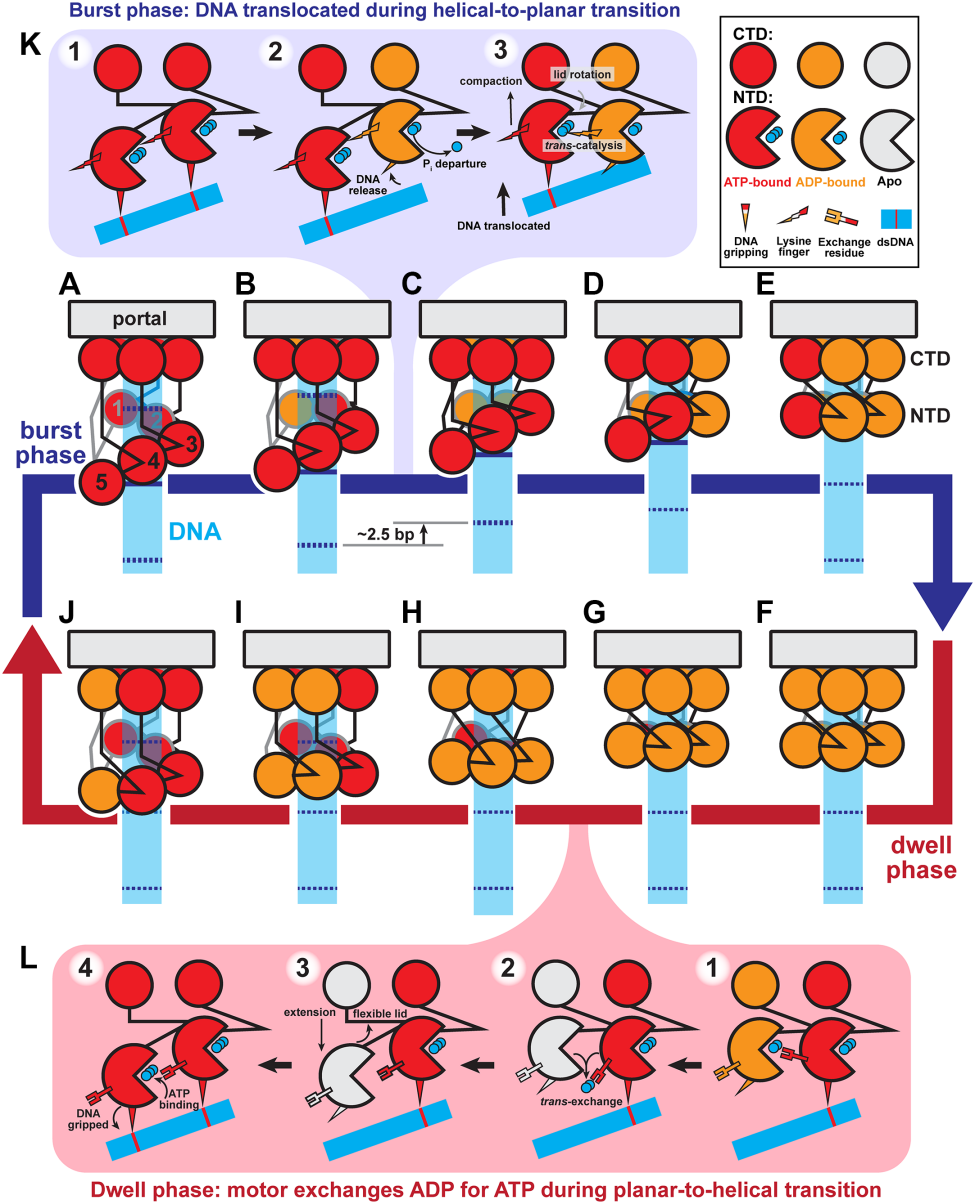
Helical-to-planar ratchet mechanism of DNA translocation. (**A–J**) Complete mechanochemical cycle. Subunits S1-S5 are labeled 1–5 in (A). During the burst phase (**A–E**), ATP-bound subunits (red) sequentially hydrolyze ATP. Hydrolysis in a subunit causes a pair of N-terminal domains (NTD) to become planar, translocating DNA. At the end of the burst, all subunits are ADP-bound (orange). During the dwell phase (**F–J**), ADP is sequentially exchanged for ATP, causing the planar NTD ring to return to the helical configuration. Helical-repeat-contacts of DNA (cyan) are marked by dashed lines. (**K**) Detailed schematic of the molecular events that coordinate the stepwise helical-to-planar transition. Initially, the two subunits’ NTD are ATP-bound, engaged with DNA, and helical. Hydrolysis and product release at the upper subunit relax its grip on DNA, allowing the other subunit's lid subdomain to rotate, bringing the NTDs into a planar configuration, translocating DNA, and aligning the two subunits for the next hydrolysis event. (**L**) Detailed schematic of the molecular events that coordinate the stepwise planar-to-helical transition. After ADP-release is promoted by the trans-acting exchange residue, the now apo subunit (white) is flexible, and can track down the helix prior to ATP-binding, which causes the subunit to engage DNA, locking the subunit in place.

Based on the results presented here, we propose a mechanism for viral DNA packaging where the motor ratchets between extended helical and compressed planar configurations to translocate DNA (Figure 7). In this model, the transition from the helical to planar states drives DNA translocation during the burst phase, while the transition back from the planar to the helical state resets the motor during the dwell phase.

#### Mechanism of force generation

As depicted in Figure 7K, ATP binding drives two competing effects. The predominant effect is increased affinity for DNA, resulting in the N-terminal domains adopting a helical configuration complementary to the helical phosphate backbone (Figure 6, Supplementary Movies S8–S12). The competing effect of ATP binding is lid subdomain rotation over the active site (Figure 5 and Supplementary Figure S5, Supplementary Movies S4–S6); because the lid subdomain is bound to a neighboring subunit (Figure 1), this effect would drive subunits into the planar configuration if not for the interaction with the helical DNA. The tension between the two effects is resolved when a subunit hydrolyzes ATP and thus releases its grip on DNA; no longer constrained by its interaction with DNA, the lid subdomain of the adjacent ATP-bound neighbor can now rotate, bringing both subunits into a planar configuration. Since the ATP-bound subunit(s) maintains grip of DNA, this results in a discrete stepping of the DNA past the hydrolyzing subunit, through the ring and into the procapsid. Thus, resolution of the competing effects of ATP binding provides the basis of force generation and DNA translocation.

#### Events in the burst phase

Our detailed description of the mechanochemical cycle starts when all five subunits are ATP-bound and therefore in the helical configuration (Figure 7A). The subunit at the top of the helix (S1) hydrolyzes first and releases its grip on DNA, allowing its ATP-bound neighbor to rotate its lid subdomain and bring both subunits into a planar configuration (transition between Figure 7A to B). Since the remaining subunits (S2–S5) have yet to hydrolyze ATP, they continue to grip DNA such that ∼2.5 bp of DNA are translocated into the procapsid. Upon planar alignment of the two N-terminal domains, the *trans*-acting lysine finger of the now-ADP-bound subunit is positioned to trigger hydrolysis in the adjacent ATP-bound subunit (Figure 7K; 2A). Hydrolysis at this subunit initiates the next translocation step, as the hydrolyzing subunit releases grip of DNA, and lid subdomain of the ATP-bound adjacent subunit rotates, bringing both subunits into plane and translocating DNA as in the previous step (transition between Figure 7B–C). This pattern of hydrolysis at one subunit coordinating force generation of the adjacent subunit permutes around the ring until all N-terminal domains are in the planar configuration (Figure 7C–E). The result of one complete helical-to-planar transition is translocation of ∼10 bp, or one helical turn, of DNA in four sub-steps. Thus, the helical-to-planar transition coordinated by sequential ATP hydrolysis constitutes the burst phase of the mechanochemical cycle.

#### Events in the dwell phase

Once the motor is in the planar configuration, the final hydrolysis event would not translocate DNA (transition between Figure 7E–F). However, hydrolysis at the final subunit (S5) is needed to release DNA, such that the motor can step back down DNA during the reset. Additionally, single-molecule studies have shown that the fifth, non-translocating hydrolysis event plays a coordination role (20). Thus, the final hydrolysis event likely coordinates the initiation of nucleotide exchange (Figure 7F). The final subunit, whose N-terminal domain is now in plane with the N-terminal domain of the subunit that began the translocation burst (S1), donates its *trans*-acting arginine exchange residue to promote nucleotide exchange in the first subunit (transition between Figure 7F–G; Figure 3, Supplementary Movie S1). Because DNA was translocated one helical turn, by DNA’s translational symmetry, the subunit that began the burst (S1) is now positioned to reengage a DNA phosphate one helical turn below its last bound position. This highlights the importance of critical electrostatic contacts every helical turn of DNA observed in SMFS experiments (65).

After ATP binds at the first subunit, it reengages DNA, locking its N-terminal domain in place. Then its *trans-*acting arginine exchange residue promotes nucleotide exchange in the adjacent subunit (S2) (transition between Figure 7G–H). Unlike the first subunit, the next subunit is misaligned with the helical DNA phosphate backbone. However, upon ADP release but before ATP binding, the subunit passes through the apo state where the lid subdomain is flexible (Figure 5). This flexibility allows the subunit's N-terminal domain to re-access the position where it is aligned with DNA (Figure 7L). Because ATP binding and DNA binding are coupled (Figure 4), this realignment would promote binding of ATP and tight binding of DNA, locking the N-terminal domain in place (62). As each subunit's N-terminal domain samples the environment in the apo state, its Walker A motif may adopt the nucleotide-blocking conformation to prevent immediate ADP rebinding or premature ATP binding (Supplementary Figure S2, Supplementary Movie S3). This process permutes around the ring until the motor resets to the five ATP-bound helical configuration that began the cycle (Figure 7H-A). Thus, the planar-to-helical transition coordinated by sequential nucleotide exchange constitutes the dwell phase of the mechanochemical cycle.

#### Initiation of the burst phase

As described above, the first hydrolysis event occurs at the sheared interface, in the subunit closest to the capsid (S1 in Figure 7A). As observed in Woodson *et al.* (19), the unique arrangement of this subunit's lid subdomain as it reaches down to maintain contact across the sheared interface in the ATP-bound helical configuration positions a glutamine in its lid subdomain close to the γ-phosphate of ATP. Hence, the glutamine may now be poised to catalyze hydrolysis *in cis*. In asccφ28, there are three potentially equivalent residues, Asn250, Lys252 and Asn254 that could function similarly. Thus, the motor likely initiates the burst phase when the ring adopts a fully helical configuration at the end of the planar-to-helical reset.

### Convergence with single-molecule results

It has been inferred from SMFS data that the homologous φ29 packaging motor couples inorganic phosphate departure to DNA translocation (22). However, this does not necessarily imply that phosphate departure provides the energy to drive the force-generating conformational changes in the motor. In our proposed model, ATP-binding creates tension between lid subdomain rotation, promoting planarity, and interactions with DNA, promoting helicity. Hydrolysis and phosphate departure resolve this tension by releasing a subunit's grip on DNA. This allows the ATP-binding energy stored within the neighboring strained lid subdomain to be converted into force exerted on the DNA. In this way, sequential phosphate departure serves as a trigger to initiate each stepwise movement of DNA.

Further, our mechanism also explains the physical basis of four translocation steps within the context of a motor with five subunits; only four steps are required to convert a pentameric helix into a planar ring. The last hydrolysis event (Figure 7E) would not translocate DNA but coordinates nucleotide exchange, consistent with SMFS (20). Additionally, the step size of the motor is set by the rise of the N-terminal domain helix and rotation of the lid subdomain, rather than the periodicity of DNA. In fact, SMFS experiments have recently shown that the motor produces three 0.85 nm sub-steps during translocation of dsDNA, DNA/RNA hybrids and dsRNA, despite varying helical parameters across these substrates (70). However, the fourth sub-step size is significantly shorter while packaging dsRNA compared to dsDNA. The abbreviated fourth sub-step enables the motor to maintain registry along the shorter helical pitch of dsRNA, again arguing that the overall burst size is coupled to realignment of the first subunit with the phosphate backbone. Thus, while the overall planar-to-helical transition is coupled to the helical pitch of the translocated substrate, the sizes of individual sub-steps depend primarily on interactions between subunits. By extension, our model thus helps rationalize how the motor translocates non-integer (2.5 bp) step sizes as has been demonstrated by SMFS (21), and accommodates sequence-dependent variability of DNA geometry.

### Comparison to models proposed for other ring ATPase systems

Two other compelling translocation models have been proposed based on reported helical structures: ‘filament treadmilling’ (68) and ‘hand-over-hand’ models (72). While there are differences between the models stemming from different geometries of FtsK-like and AAA+ assemblies, the basic proposed translocation mechanism is the same. In both cases, ATP hydrolysis and product release at one end of the ATPase domain helix cause that subunit to disengage from the biopolymeric substrate. Subsequent ATP binding at the other end of the helix causes that subunit to engage the substrate. The net result is translocation of the biopolymer relative to the spiral.

A defining feature of φ29-like DNA packaging mechanochemistry is the biphasic burst-dwell cycle (20,21) which is incompatible with both the filament treadmilling and hand-over-hand models. Because each step is rotationally symmetric to the previous step, these models are inherently continuous with no obvious symmetry breaking required to begin or end the burst phase. Another asymmetric feature is the presence of a non-translocating hydrolysis event (20,21). In contrast, these key features naturally emerge from our helical-to-planar ratchet model. Compaction of the ring from the helical to planar configuration is the translocation burst (Figure 7A–E), while extension of the ring from the planar to helical configuration is the nucleotide-exchange dwell (Figure 7F–J). The last hydrolysis event when the motor is planar cannot translocate DNA, thus explaining a non-translocating hydrolysis event. This type of mechanism may also explain the burst-dwell behavior of the polypeptide-translocating ClpX proteosome machinery (73) and can serve as a general framework for any ring systems that are shown to exhibit burst-dwell dynamics.

Nonetheless, despite the differences in overall mechanisms, there seem to be similarities in the core components. To summarize: in all cases, ATP hydrolysis causes a subunit to disengage from the translocated substrate, substrate is translocated by the remaining ATP-bound subunits, and rebinding of ATP causes subunits to reengage substrate. Further, in both our helical-to-planar ratchet and the hand-over-hand model, the repositioning of subunits is accomplished by rotation of the lid subdomain of a subunit coupled to ATPase activity. Thus, these alternative models could be considered as variations of an underlying mechanistic theme.

## DATA AVAILABILITY

The structure determined from the trigonal space group is deposited as PDB ID 7JQ6; the native and sodium iodide structures from the tetragonal space groups are deposited as PDB ID 7JQP and 7JQ7, respectively.

## Supplementary Material

gkab372_Supplemental_Files
