# Supplementary material for: Atomistic basis of force generation, translocation, and coordination in a viral genome packaging motor
Source: Nucleic Acids Res. 2021 May 29;49(11):6474–88. doi: 10.1093/nar/gkab372 (PMC8216284; doi:10.1093/nar/gkab372)
Supplement: gkab372_Supplemental_Files [file gkab372_supplemental_files.zip › phi28_SI_NAR.docx]

| **Crystal** | **Se-Met** | **NaI** | **Native** |
| --- | --- | --- | --- |
| PDB-ID | 7JQ6 | 7JQ7 | 7JQP |
| Source | 21-ID-F | 21-ID-G | 21-ID-F |
| Wavelength (Å) | 0.97872 | 0.97857 | 0.97872 |
| Detector | Rayonix MX300 | Rayonix MX300 | Rayonix MX300 |
| Spacegroup | P3_2_21 | P4_3_2_1_2 | P4_3_2_1_2 |
| Resolution (highest shell) | 2.8 (2.85-2.80) | 2.9 (2.95-2.90) | 2.9 (2.95-2.90) |
| **Cell Dimensions** |  |  |  |
| a (Å) | 135.0 | 112.2 | 110.9 |
| b (Å) | 135.0 | 112.2 | 110.9 |
| c (Å) | 276.7 | 354.2 | 351.8 |
| Α, β, γ (°) | 90 90 120 | 90 90 90 | 90 90 90 |
| No Frames | 720 | 234 | 240 |
| Osc Range (°) | 1 | 0.5 | 0.5 |
| No. Reflections | 5,302,794 | 2,924,141 | 3,203,867 |
| No. Merged Reflections (Anom) | 43,588 (62,234) | 42,759 (78,494) | 49,847 |
| Rpim | 0.028 (1.4) | 0.04.3 (1.1) | 0.05.7 (1.0) |
| CC1/2 | NA (0.52) | 0.99 (0.21) | 0.98 (0.30) |
| I/s | 16.8 (0.5) | 16.3 (0.6) | 11.0 (0.6) |
| Completeness (%) | 85 (31) | 99.7 (97) | 98.9 (90) |
| Redundancy | 43 (12) | 16.4 (7.5) | 10.9 (4.8) |
| **Refinement** | **Se-Met** | **NaI** | **Native** |
| **Phasing** | SAD | MR | MR |
| Resolution (highest shell) | 2.9 (2.94-2.90) | 2.9 (2.93-2.90) | 3.0 (3.03-3.00) |
| No. Unique Reflections | 43,583 | 42,759 | 38,744 |
| Rw (%) | 24.12 | 23.46 | 23.80 |
| Rf (%) | 27.41 | 28.37 | 27.31 |
| Bond (Angle) RMS (Å)(°) | 0.004 (0.6) | 0.004 (0.7) | 0.004 (0.9) |
| No. Molecules in a.u. | 5 | 5 | 5 |
| Non-hydrgon protein atoms avg. B (N) | 93 (15,401) | 58 (15,123) | 56 (15,065) |
| Non-hydrogen  solvent atoms avg. B (N) | NA (0) | 81 (152) | 41 (31) |
| **Ramachandran** |  |  |  |
| Favoured (%) | 97.80 | 97.36 | 97.22 |
| Allowed (%) | 2.20 | 2.48 | 2.73 |
| Outliers (%) | 0.00 | 0.17 | 0.06 |

**Table S1: Crystallographic Data and Refinement Statistics**


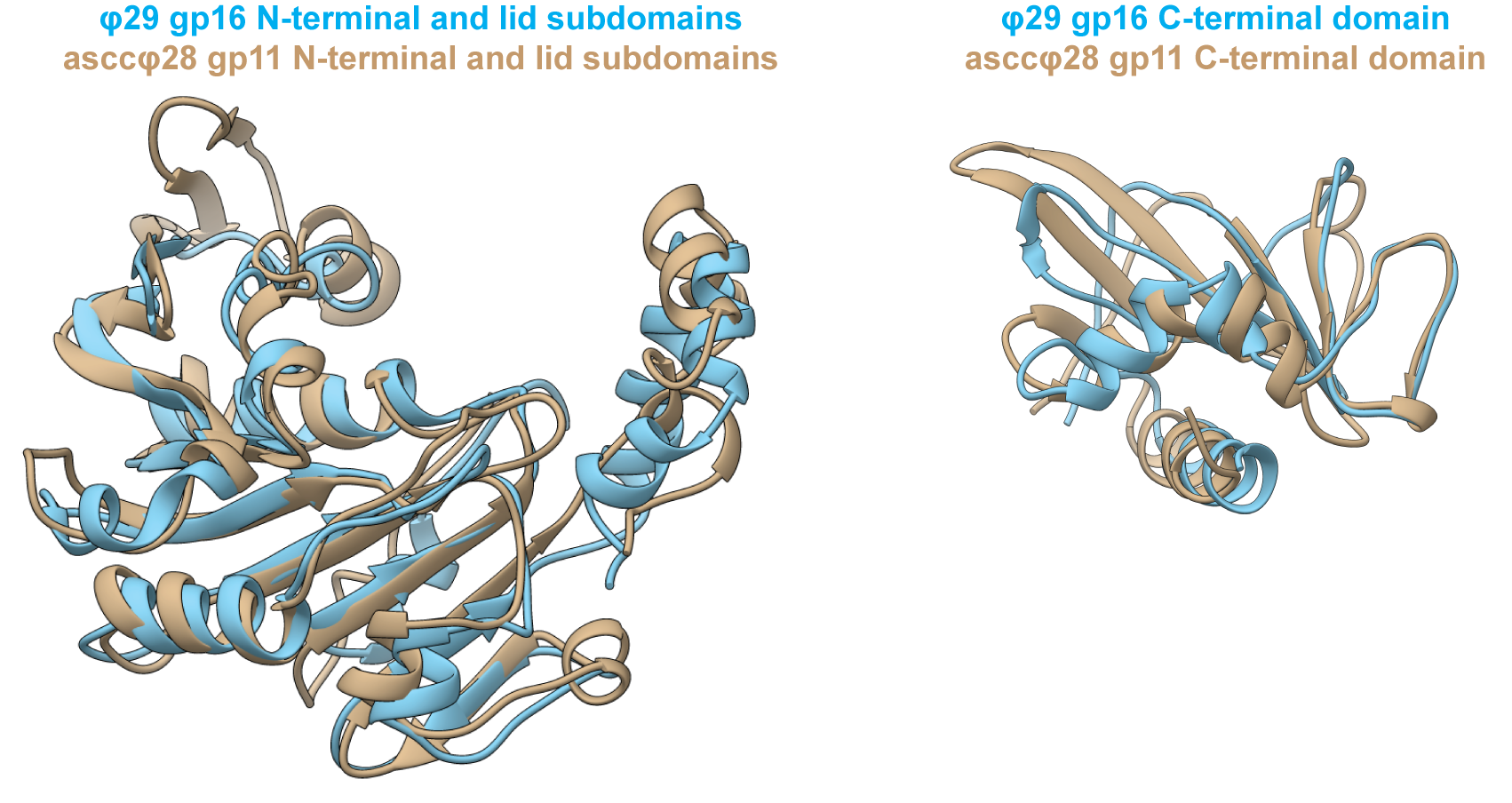


**Figure S1:** Superimpositions of the φ29 gp16 and asccφ28 gp11 domains show nearly identical structures, as expected based on their 45% sequence similarity.


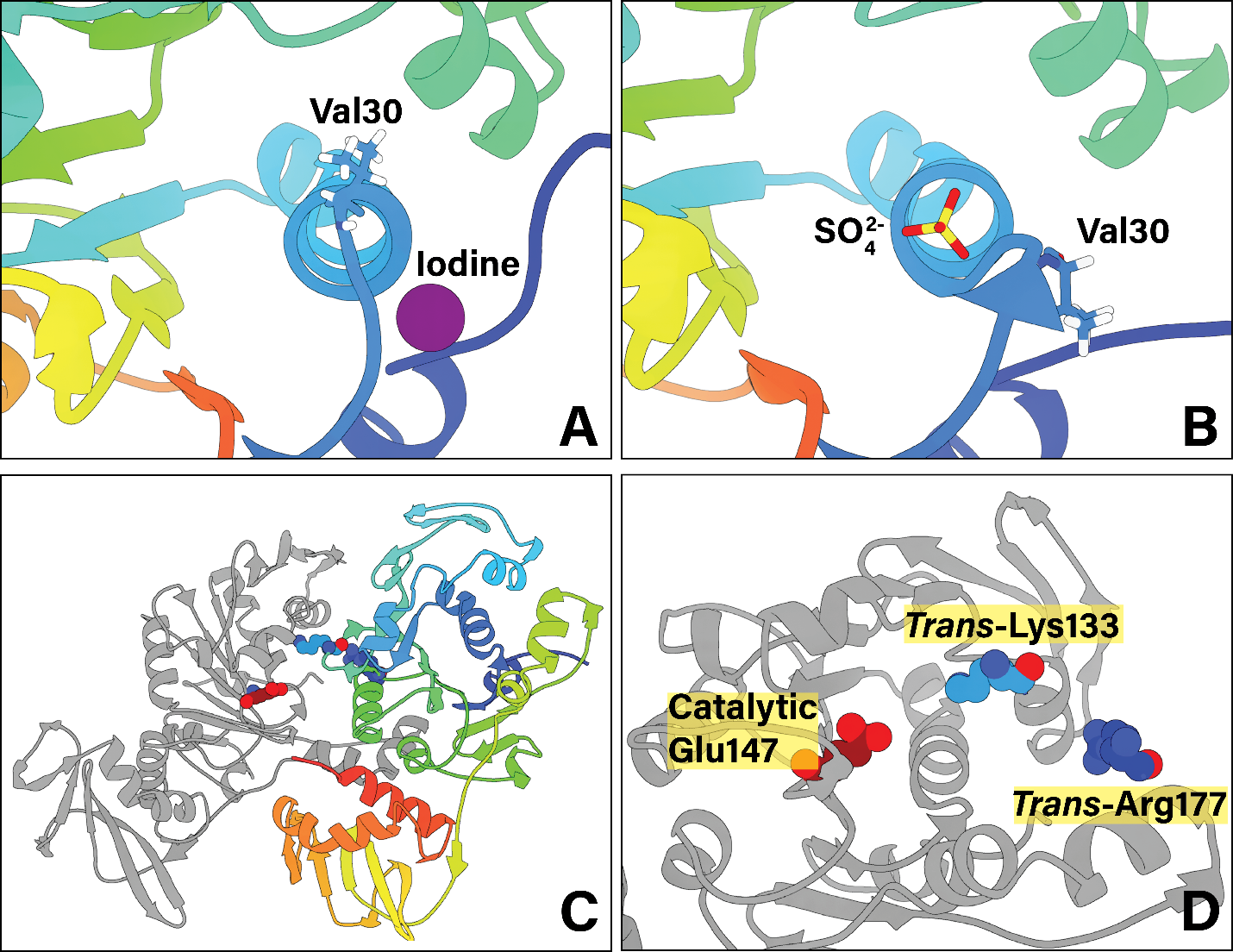


**Figure S2: The active site observed in the crystal structure.** Two crystals show that the Walker A motif varies helical content. Extra helical content (**A**) places Val30 in the binding pocket occluding nucleotide occupancy, and less helical content (**B**) points Val30 away from the binding pocket. This allows a sulfate ion to mediate interactions in the P-loop normally mediated by the β-phosphate of ATP/ADP. (**C**) A zoomed-out view of two neighboring subunits, shown in gray and rainbow. (**D**) Zoom in on the active site shows that the trans-acting Lys133 is considerably closer to the cis-acting catalytic Glu147 than trans-acting Arg177 is; from this alone it can be inferred that the lysine acts as a lysine finger, as it is better situated to interact with the γ-phosphate of ATP near the catalytic Glu147.


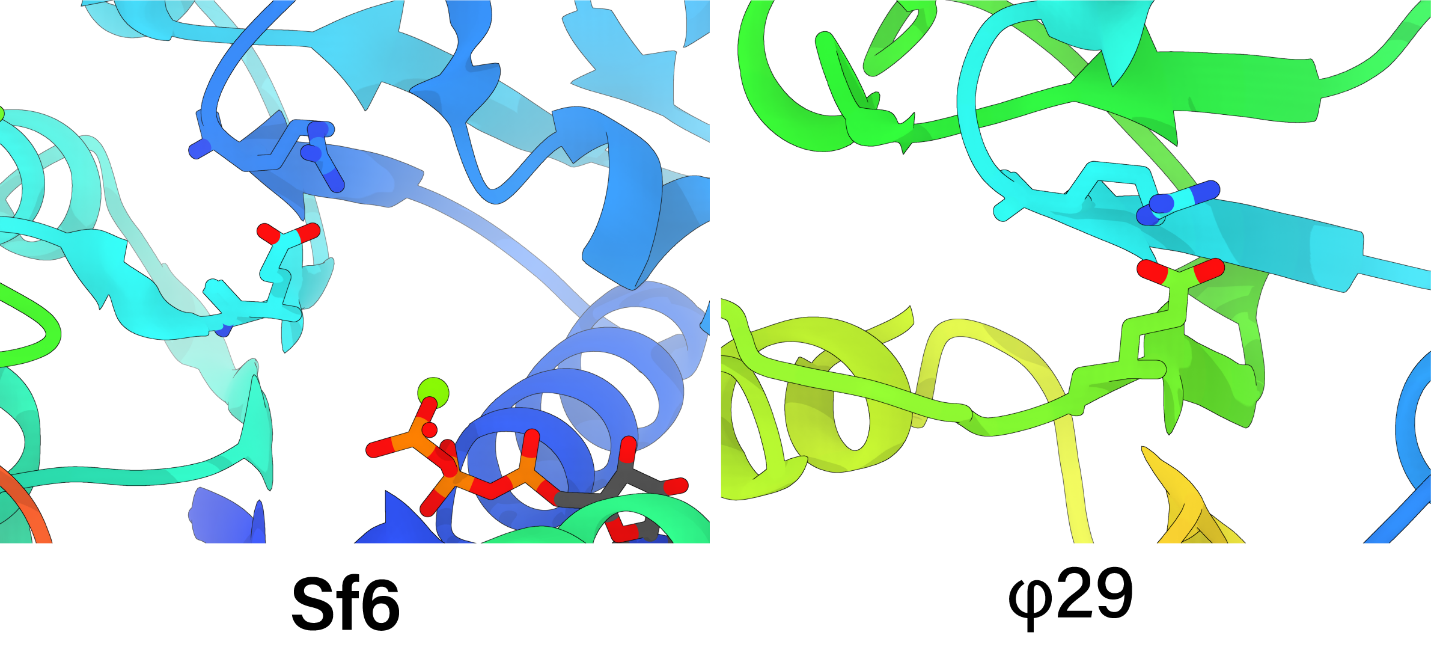


**Figure S3: The catalytic glutamate is observed to be inactive in the Sf6 and φ29 crystal structures.** The Sf6 and φ29 packaging ATPases are shown as Richardson diagrams, and zoomed into the ATPase active site. The catalytic glutamate is shown as sticks, pointing its carboxylate away from ATP and towards a conserved arginine residue also shown as sticks, reminiscent of the glutamate switch mechanism found in other AAA+ enzymes.


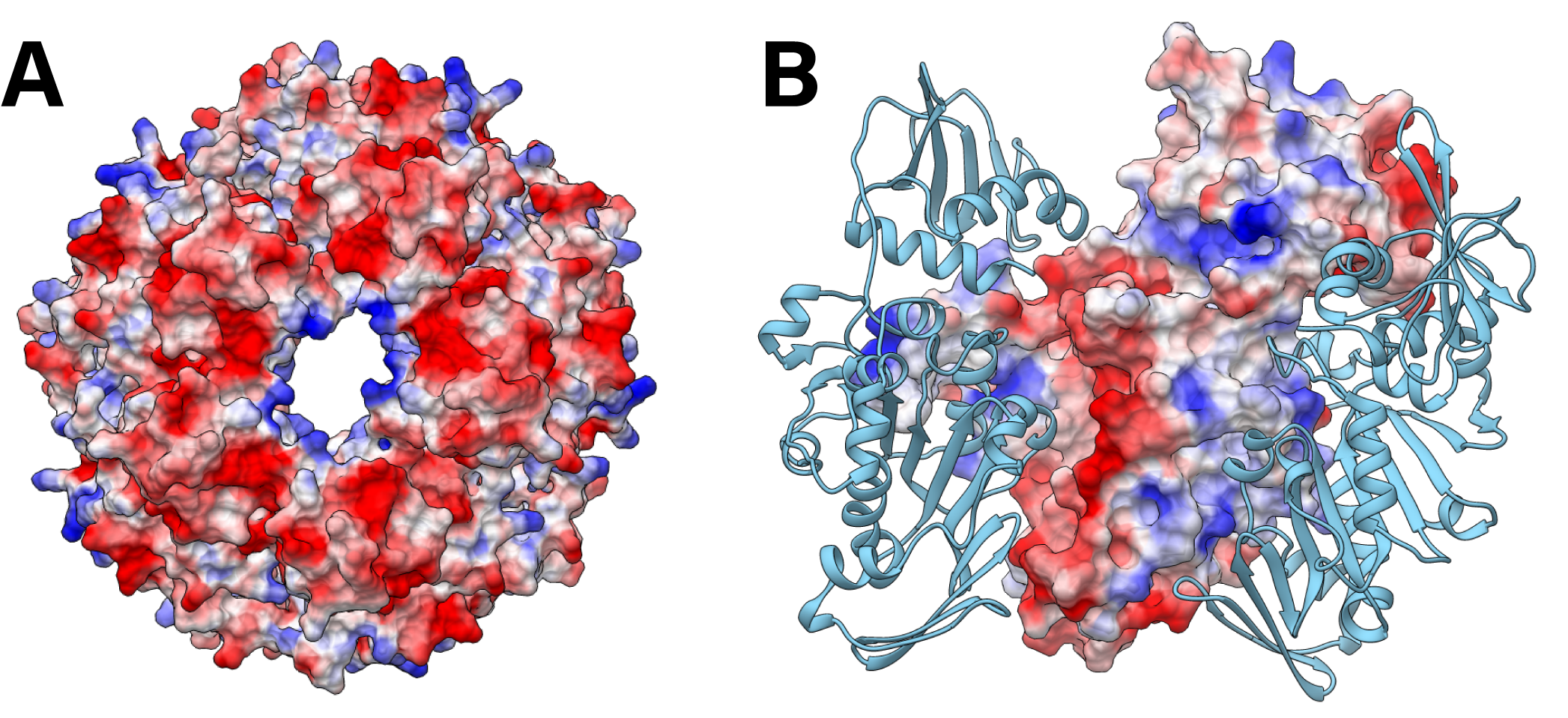


**Figure S4: Electrostatics of the pore.** (**A**) An end-on view of the gp11 pentamer crystal structure shows that the interior of the pore is lined with positive electrostatic surface potential. (**B**) A cut away view of the pentamer shows a positive band of electrostatic potential runs along the length of the monomer, creating a DNA-gripping strip. Two neighboring subunits are shown as cyan Richardson diagrams.

| **phi28** | **Lys66** | **Lys92** | **Lys107** | **Arg110** | **Arg128** |
| --- | --- | --- | --- | --- | --- |
| **phi29** | n/a | n/a | Lys81 | Arg83 | Lys56 |
| **Sf6** | Arg63 | n/a | Gln81 | Arg82 | Asn101/Asn102 |
| **T4** | Lys204 | Lys83/Arg84 | Lys94/Arg95 | Lys223 | n/a |
| **P74-26** | n/a | n/a | n/a | Arg101 | Arg132 |
| **D6E** | Lys87/Arg88 | Arg96 | Lys98 | Arg101 | Lys123 |

**Table S2: Conservation of positively charged residues located in the pore.** The top row highlights positively charged residues found in the pore of the asccφ28 packaging ATPase, situated such that they can interact with substrate DNA. Superimposing solved ATPases onto the asccφ28 structure, residues proximal in cartesian space (not necessarily sequence space) are identified. If no residue is proximal, the cell is shaded red, if a polar residue is proximal the cell is shaded orange, and if a positively charged residue is proximal, the cell is shaded yellow. In some cases, more than one residue is proximal, and the cell has two residues labeled.


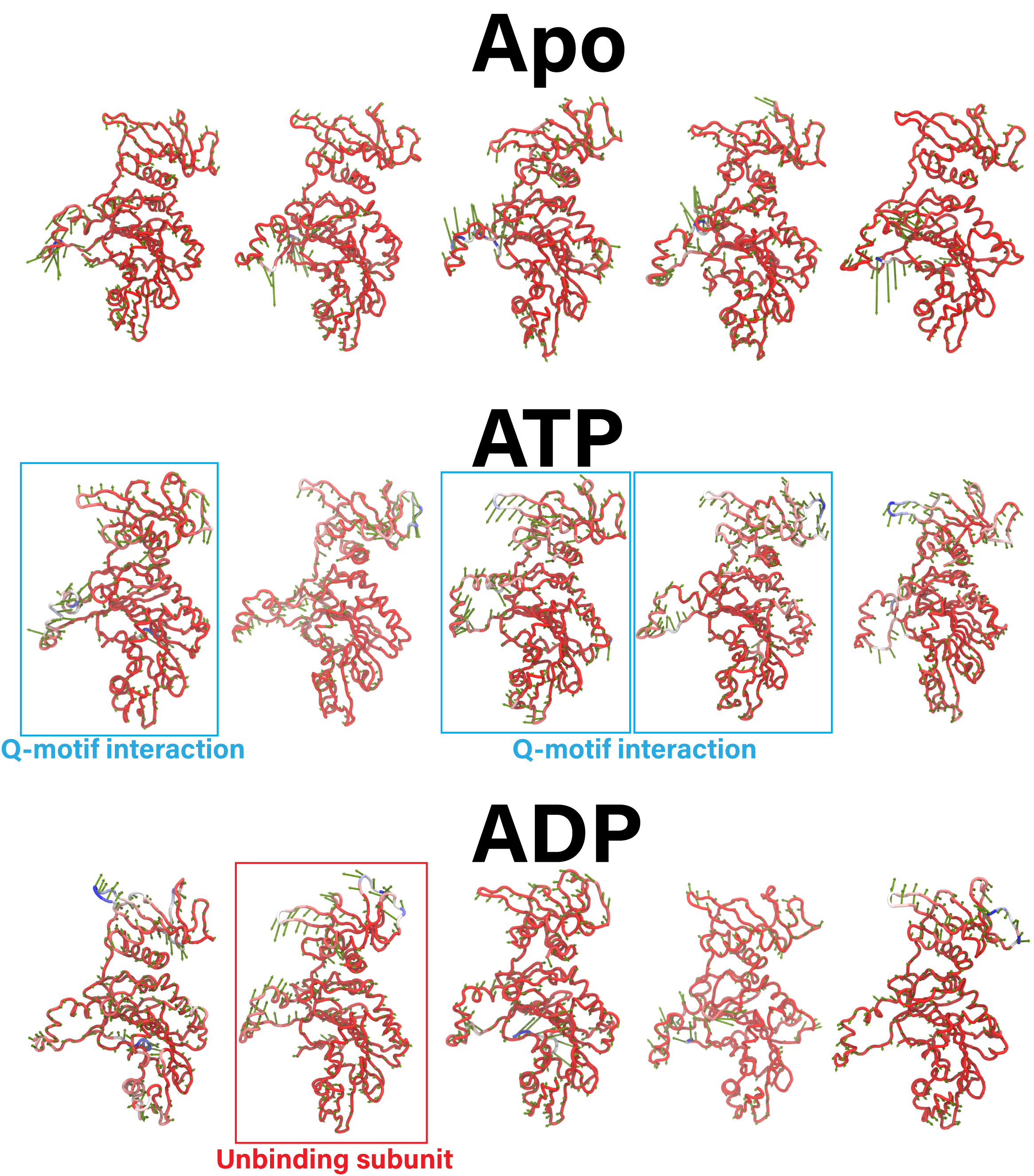


**Figure S5: the first principal component of alpha-carbon motion for all five subunits in the apo, ATP-bound, and ADP-bound states.** We find that the lid subdomain of the apo state has seemingly no correlation, suggesting that stochastic fluctuations dominate. In the ATP-bound state, the lid subdomain rotates towards the ATPase active site, particularly evident in subunits which maintain the Q-motif interaction with the adenosine base. In the ADP-bound state, the lid subdomain of the subunit which unbinds ADP rotates away from the ATPase active site. This motion is also highlighted in Movie S2. These principal components are evidence that nucleotide occupancy and binding actuate lid subdomain rotation.


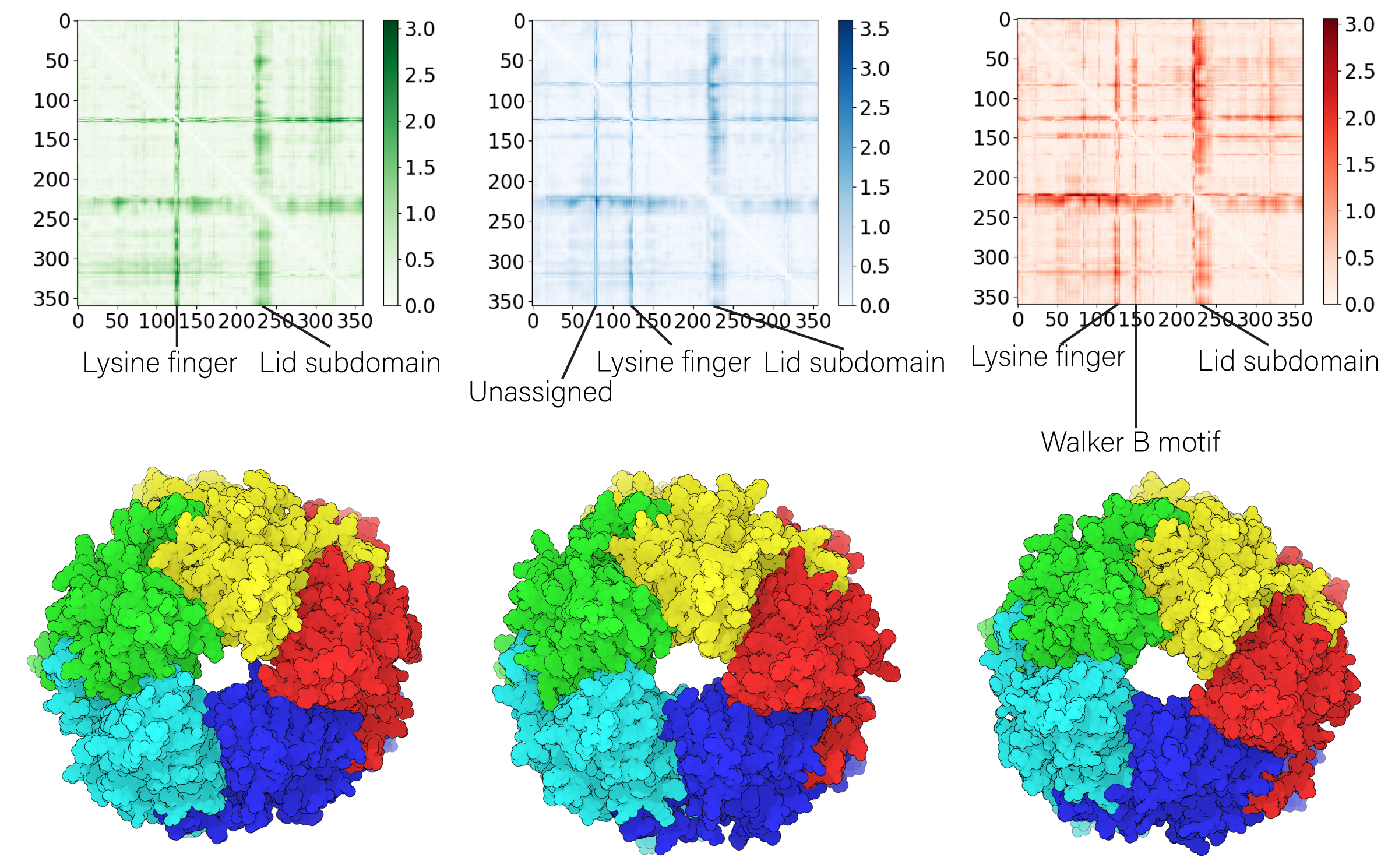


**Figure S6: variability in crystal structures.** (**Top row**) The three solved crystal structure show large standard deviations in positions of the lid subdomain and trans-acting lysine finger. This indicates that the lid subdomain rotation can be used to position the lysine finger of a neighboring subunit. (**Bottom row**) the crystals are not regular pentagonal structures, rather, they are oblong. This indicates that deviations in the lid subdomain, which mediated most inter-subunit contacts, can be used to change the structure’s quaternary structure.


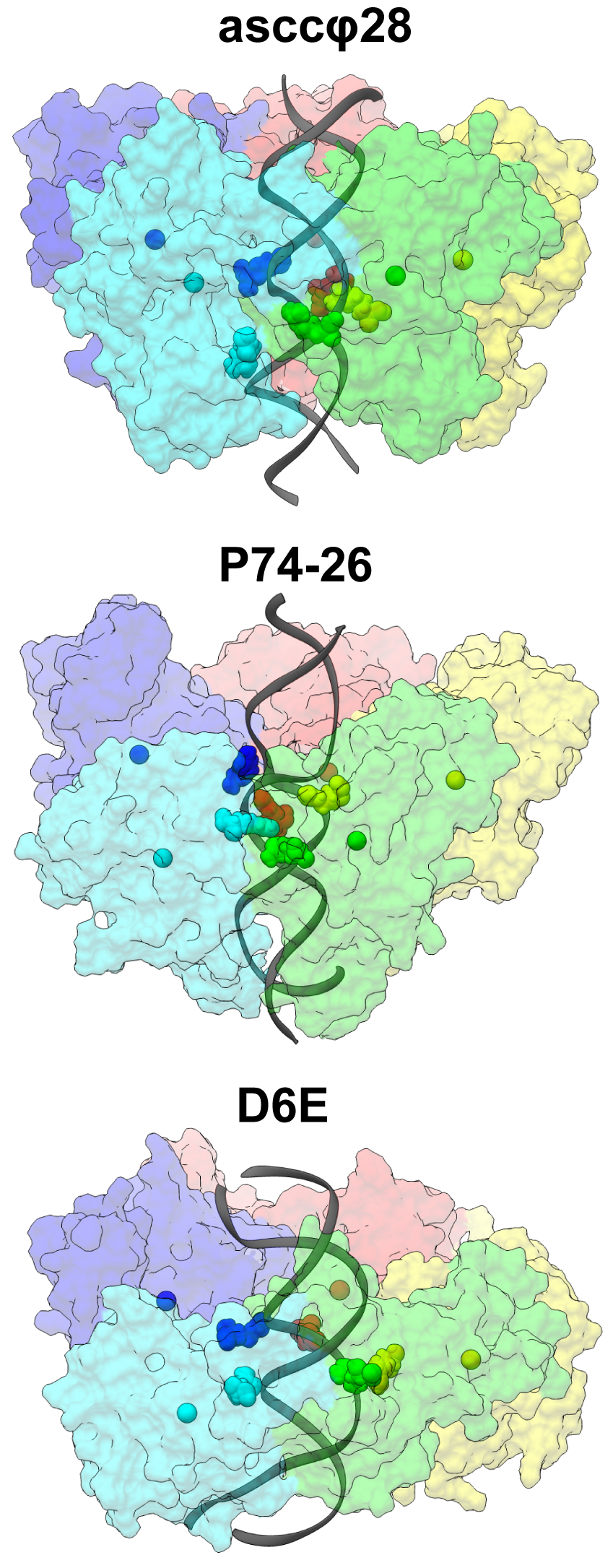

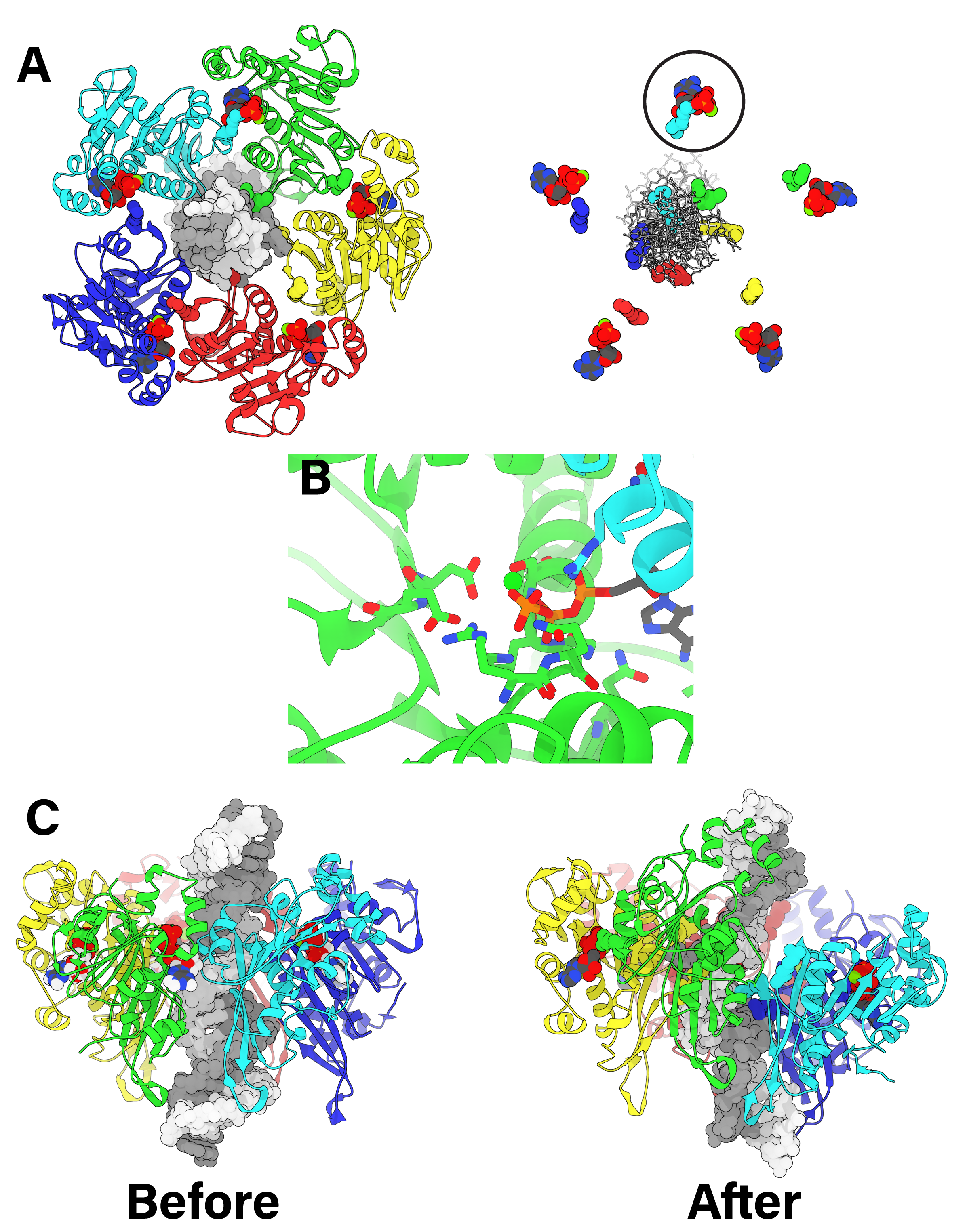


**D**

**Figure S7: P74-26 pentamer model and simulations.** (**A**) The pentamer in the presence of ATP and substrate DNA. Arg101 binds to DNA in the pore, and Arg139 interacts in trans to coordinate the γ-phosphate of ATP. These agree with biochemical data showing Arg101 is necessary to grip DNA, and Arg139 catalyzes hydrolysis in trans. On the right these interactions are shown in isolation for easier visualization. The binding pocket where Arg139 is best positioned to catalyze hydrolysis is circled. (**B**) Zooming in on a single binding pocket, where Arg139 is best positioned to catalyze ATP hydrolysis, we find that all the expected interactions are maintained based on prior monomer simulations and ATPase enzymology. (**C**) The ATP at this active site was removed and the structure was simulated in a 4-ATP-bound, 1-apo state. At the apo interface, the pentamer shears to form a helical pitch, highlighted in this before and after view. (**D**) The predicted helical arrangements of the P74-26 and D6E packaging ATPases are depicted. Each subunit is shown as a transparent surface. The centers of mass of each subunit are depicted as hard spheres to show the right-handed helical arrangement descending from the blue to cyan subunits. DNA-gripping residues are shown as spheres, to show their coordination with the substrate DNA, whose phosphate backbone is a dim gray ribbon.

**Movie S1: ADP-release by the exchange residue (ER).** ADP-release is characterized by dissociation from the cis-acting Walker A lysine and association to the trans-acting EF arginine. The cis­-acting enzyme is shown in light blue, and the trans-acting enzyme is shown in green. Bound Mg^2+^-ADP, cis-lysine, trans-arginine, as well as residues which interact with adenosine are shown in spheres. The enzyme keeps contact with the adenosine, using the bound base as a fulcrum to pull out the phosphates.

**Movie S2: P-loop backbone conformation change upon ADP release.** As ADP is released from the subunit, the P-loop backbone dihedral angles rotate to position a backbone oxygen atom close to where the β-phosphate was prior to release.

**Movie S3: The Walker A Val30 becomes helical in absence of bound nucleotide.** In the mixed-occupancy 4-ADP-bound, 1-apo simulation, the apo enzyme Walker A motif adopts a more helical conformation. This places Val30 in the “blocking” pose observed in the iodine-derivatized crystal. This suggests that the Walker A conformation observed in this crystal is not merely an artifact of iodine-binding, but may serve a regulatory role in ADP-release and ATP-binding.

**Movie S4: Dynamics of apo monomer.** A 100-ns apo monomer simulation with the AMBER99SB-ILDN/TIP3P protein/water force field pairing. The enzyme is shown with a surface representation, and the lid subdomain is highlighted in yellow. The lid subdomain remains far from the active site and dynamic in the monomer apo simulations. This monomer result helps rationalize the observed flexibility of the lid subdomain in the apo pentamer simulation (see main text **Fig. 5**).

**Movie S5: Dynamics of ATP-bound monomer.** A 100-ns ATP-bound monomer simulation with the AMBER99SB-ILDN/TIP3P protein/water force field pairing. The enzyme is shown with a surface representation, and the lid subdomain is highlighted in yellow. Bound Mg^2+^-ATP are shown as spheres. The lid subdomain rotates and closes over the binding pocket, as is expected of a lid subdomain in the ASCE superfamily. This is consistent with prior MD simulations and solved crystal structures. This monomer result helps rationalized the observed rigidity of the lid subdomain in the ATP-bound pentamer simulation (see main text **Fig. 5**). This lid subdomain rotation is proposed to be the force-generating mechanism that translocates DNA (see main text **Fig. 7**).

**Movie S6: Dynamics of ADP-bound monomer.** A 100-ns ADP-bound monomer simulation with the AMBER99SB-ILDN/TIP3P protein/water force field pairing. The enzyme is shown with a surface representation, and the lid subdomain is highlighted in yellow. Bound Mg^2+^-ADP are shown as spheres. The lid subdomain rotates and closes over the binding pocket, as is expected of a lid subdomain in the ASCE superfamily. This is consistent with prior MD simulations and solved crystal structures. This monomer result helps rationalized the observed rigidity of the lid subdomain in the ADP-bound pentamer simulation (see main text **Fig. 5**). A rigid lid subdomain helps subunits which have already hydrolyzed act as a fulcrum for subunits which have not; this helps subunits not be pulled away from the viral capsid by neighboring lid subdomain rotation (see main text **Fig. 7**).

**Movie S7: D6E monomer simulation positions the lid subdomain to mediate inter-subunit contacts.** During a 2.4 microsecond simulation of the D6E monomer ATPase, the lid subdomain (yellow) extends away from the ATPase domain (green). The movie shows an interpolation between the crystal structure and the predicted extension. A gray subunit is placed by aligning both subunits to the gp11 pentamer. The interpolation shows that extending the lid subdomain would help facilitate inter-subunit contacts, akin to the gp11 pentamer.

**Movie S8: Helical-to-planar morph.** A morph between the asccφ28 helical configuration predicted from MD simulations and the starting planar structure. The DNA-gripping Arg110 are shown as spheres, and the helical phosphate backbone of DNA is shown as ribbons.

**Movie S9: Helical-to-planar morph.** A morph between the asccφ28 helical configuration predicted from MD simulations and the starting planar structure. The DNA-gripping Arg110 are shown as spheres, and the helical phosphate backbone of DNA is shown as ribbons. Each subunit is shown as Richardson diagrams.

**Movie S10: asccφ28 gp11 ATPase domain pentamer adopts a helical structure and fits into the φ29 cryo-EM reconstruction.** MD simulation of the asccφ28 gp11 pentamer ATPase domains in the 5-ATP-bound configuration predicts that the pentamer adopts a helical structure as the subunits track DNA. This pentamer fits well into the helical φ29 asymmetric cryo-EM reconstruction.

**Movie S11: P74-26 ATPase domain pentamer adopts a helical structure and fits into the φ29 cryo-EM reconstruction.** MD simulation of the P74-26 pentamer ATPase domains in the 4-ATP-bound, 1-apo configuration predicts that the pentamer adopts a helical structure as the subunits track DNA, sheared at the apo-interface. This pentamer fits well into the helical φ29 asymmetric cryo-EM reconstruction.

**Movie S12: D6E ATPase domain pentamer adopts a helical structure and fits into the φ29 cryo-EM reconstruction.** MD simulation of the D6E pentamer ATPase domains in the 5-ATP-bound configuration predicts that the pentamer adopts a helical structure as the subunits track DNA. This pentamer fits well into the helical φ29 asymmetric cryo-EM reconstruction.
